# Quantitative Ocular Motor / Vestibular Assessment in Patients with Spinocerebellar Ataxia Type 3 (SCA3, Machado Joseph Disease) – Systematic Review of the Literature

**DOI:** 10.1007/s12311-026-02021-2

**Published:** 2026-05-12

**Authors:** Ines Melzer, David J. Szmulewicz, Alexander Andrea Tarnutzer

**Affiliations:** 1 Faculty of Medicine, University of Zurich, Zurich, Switzerland; 2 Balance Disorders and Ataxia Service, Royal Victoria Eye and Ear Hospital, East Melbourne, Melbourne, VIC 3002 Australia; 3 The Bionics Institute, East Melbourne, Melbourne, VIC 3002 Australia; 4 Neurology Cantonal Hospital of Baden, Im Ergel 1, Baden, 5404 Switzerland

**Keywords:** Hereditary ataxia, Eye movements, Quantitative analysis, Vestibular, Oculography

## Abstract

Spinocerebellar ataxia type 3 (SCA3 or Machado-Joseph disease) is the second most common autosomal-dominant hereditary ataxia and often presents with ocular motor and vestibular impairment. The value of ocular motor testing for early disease detection, monitoring disease progression and as a potential treatment biomarker has been acknowledged in several studies. This systematic review aims to summarise the current evidence supporting quantitative eye movement testing in SCA3. Studies reporting on quantitative ocular motor and/or vestibular measurements in SCA3 were searched for in MEDLINE and Embase. Thirty studies reporting on 432 SCA3 patients (including 64 pre-symptomatic carriers) were included. Information on various ocular motor parameters were extracted and correlations with other parameters (including clinical scales, imaging, and genetic testing) were assessed. Ocular motor abnormalities observed comprised (horizontal) pursuit eye-movement (PEM) gain reduction (14/19 patients), saccadic intrusions (principally square-wave jerks (SWJ, 29/45)), gaze-evoked nystagmus (GEN, 65/82), and rebound nystagmus (8/10). Additionally, saccades to target displayed increased latency (13/29), reduced peak velocity (16/49) and dysmetria (33/49). Vestibular deficits measured included vestibulo-ocular reflex (VOR) gain-reductions during quantitative head-impulse testing (qHIT, 117/133) and abnormal responses during caloric irrigation (7/9), impaired visually-enhanced VOR-responses (5/6), and impaired cervical (19/66) and ocular (10/14) vestibular-evoked myogenic-potential testing. In summary, impaired VOR-gains (qHIT), GEN, horizontal PEM-gain decreases, dysmetric saccades, and SWJ were most frequently reported and therefore seem most suitable as potential disease markers. The quantitative ocular motor/vestibular assessment may facilitate early diagnosis, distinction of disease stages and also potentially allow monitoring of disease progression and treatment response.

**Supplementary Information:**

The online version contains supplementary material available at https://doi.org/10.1007/s12311-026-02021-2.

## Introduction

Spinocerebellar ataxia type 3 (SCA3), also known as Machado-Joseph Disease (MJD) is the second most common dominantly inherited cerebellar ataxia. Its prevalence is estimated at 1:50’000–100’000 people [1]. Ataxin-3 is a deubiquitinase that normally regulates protein homeostasis [2, 3]. In SCA3 the CAG trinucleotide repeat expansion leads to production of an abnormally elongated polyglutamine (polyQ) tract in ataxin-3, causing misfolding which in turn leads to ataxin-3 protein aggregation [4–6]. Aggregation of mutant ataxin-3 is further facilitated by proteolytic cleavage into toxic fragments enriched in the polyQ region [7]. The misfolded proteins tend to accumulate as intranuclear and cytoplasmic inclusions, often associated with ubiquitin and proteasome components [5, 8]. In healthy individuals ataxin-3 is a cytoplasmatic protein [9]. However, in SCA3 patients, it is also located in the nucleus, where the aforementioned aggregates are a neuropathological hallmark of the disease [10]. As a result, neuronal proteostasis, transcriptional regulation, and mitochondrial function become progressively impaired, leading to selective neurodegeneration primarily in the cerebellum, brainstem, basal ganglia and spinal cord [5, 6, 9].

Disease age of onset varies from 5 to 70 years [11] and is inversely correlated with CAG repeat length [12]. In one large study of 412 people from 82 families with SCA3, a mean survival time of 64 years was reported (compared to 79 years in an unaffected comparator cohort). A subset of 366 patients was further analysed to reveal a mean age at disease onset of 36 years and survival following disease onset was an average of 21 years. These values were largely mediated by CAG repeat length [13]. Mortality most often results from aspiration pneumonia in the context of dysphagia, respiratory infections and secondary sepsis [14–16]. Less commonly, “direct brainstem involvement” contributes to death via bulbar failure and respiratory compromise from underlying severe neuronal loss in respiratory and motor nuclei [17]. SCA3 remains an uncurable and often fatal disease within 20–25 years after symptom onset [18]. However, efforts are being made at developing future treatments, e.g. gene therapy, stem cell therapy, genome editing, and more conventional medication modalities [19].

Clinically, SCA3 is characterized by a progressive cerebellar syndrome in combination with a variable range of extracerebellar features including: pyramidal signs (spasticity, hyperreflexia), extrapyramidal manifestations (dystonia, parkinsonism), lower motor neuron involvement, a sensory ganglionopathy and vestibular hypofunction [20]. Non-motor symptoms such as fatigue, pain, sleep disturbance, autonomic dysfunction (e.g., urinary or orthostatic hypotension), significantly contribute to disease burden [21]. Ocular motor abnormalities may be the observed in early stages of the disease [22–24], highlighting their role as valuable biomarkers in early differential diagnosis, including during pre-symptomatic stages. Despite this, and in difference to *bedside* ocular motor testing, observations from *quantitative* testing are scarce, due to a combination of: (1) limited overall published data, and (2) where published, a narrow range of ocular motor and/or vestibular domains studied.

To address these limitations, we systematically reviewed the literature on quantitative ocular motor and vestibular abnormalities in SCA3 with the goal to identify a set of ocular motor/vestibular parameters that may be quantitatively assessed in an effort to facilitate narrowing the differential diagnosis prior to genetic testing (where available), application in natural history studies, and biomarker use in treatment trials. Furthermore, we set out to assess potential correlations between ocular motor and vestibular findings, and other parameters, including disease onset, duration, and severity in order to identify those examinations most suitable for these various applications.

## Methods

### Data Sources and Searches

MEDLINE (PubMed) and Embase were searched for original articles reporting on quantitative ocular motor and vestibular abnormalities in SCA3 using a search string that defined the clinical syndrome and narrowed the obtained literature to studies reporting on ocular motor or vestibular features and quantitative assessments. Additionally, all citations of selected articles were searched manually, and corresponding authors were contacted for additional information if needed. Further details are provided in the online Appendix 1. As a systematic review, ethical approval was not required for this work. We did not register this systematic review on PROSPERO.

## Study Selection and Quality Assessment

**Fig. 1:**
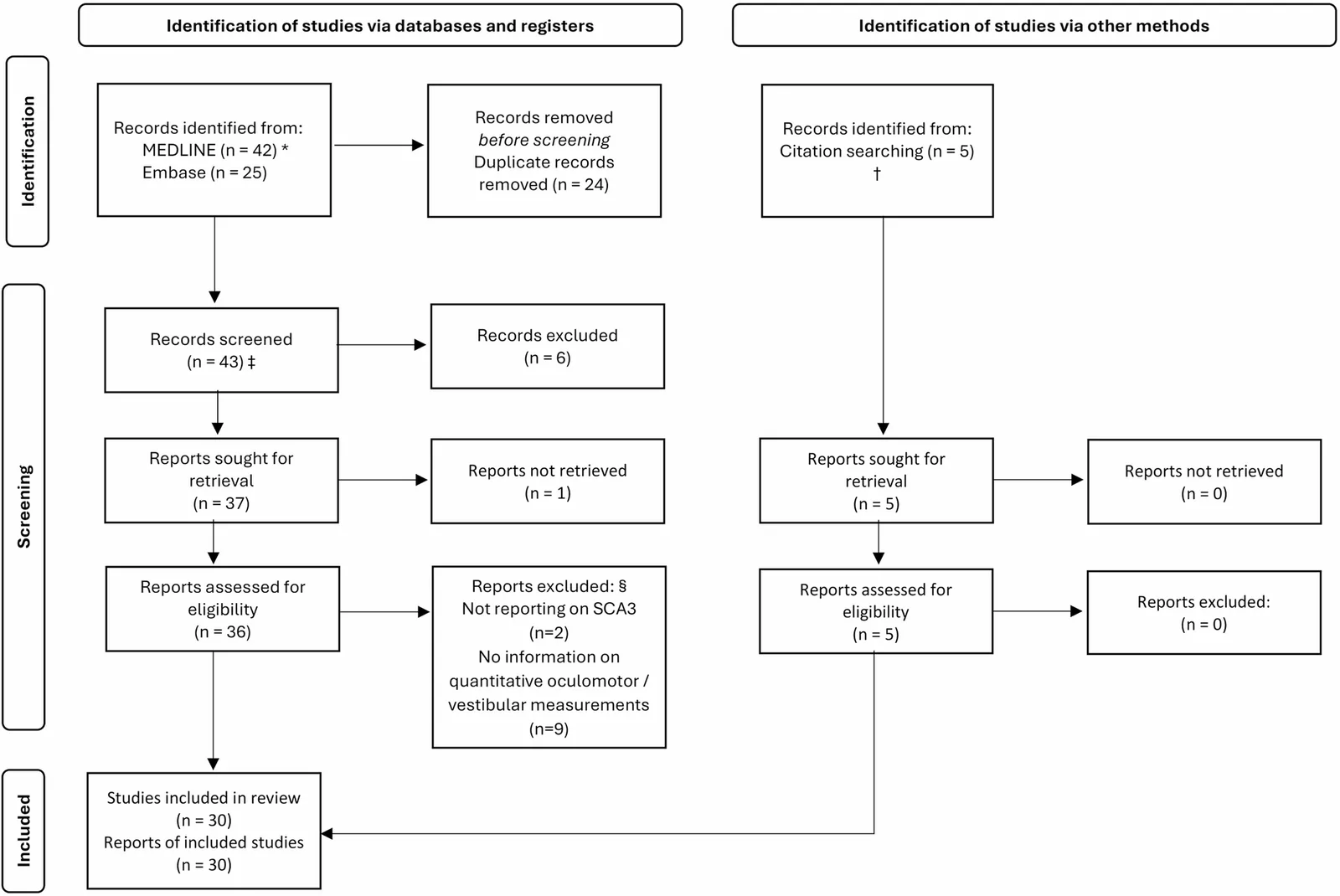
**PRISMA Flow Chart** [78]. * MEDLINE was accessed via PubMed. † Five manuscripts in total were identified through hand search. ‡ Abstracts coded as “yes” or“maybe” by at least one reviewer were included in full-text review. § If initial full text evaluation by two independent reviewers (AAT, IM) differed, consensus was reached through discussion

Two reviewers (IM, AAT) screened all citations. Only studies in English that included original quantitative data on genetically confirmed SCA3-patients were considered (see Fig. 1 - PRISMA flow-chart). Initially, a title and abstract review was performed. All articles selected by one or both reviewers were then reviewed in full text as a second step. After agreeing on full-text inclusion, the inter-rater agreement was calculated with Cohen’s kappa [25]. The quality of the included studies was rated based on the following criteria: patient and control group selection; recording protocol, devices and parameters; and data analysis and significance; according to a previous publication [26]. Subsequently an overall study-quality rating was determined (high, moderate, low; see also Appendix 2).

We identified overlap of study cohorts in several publications. The same study cohort was examined in two publications [27, 28]. Furthermore, there were study cohort overlaps in another two studies [29, 30]. Additional overlaps of these two [29, 30] and other publications [31–34] as well as probable overlaps in [35] and [36], and [37] and [38] were enquired with the corresponding authors, but could neither be confirmed nor rejected.

## Data Extraction, Synthesis, and Analysis

Information extracted in all selected studies included the study design, epidemiologic details of the participants, parameters assessed and recording methods used. We searched for details on ocular motor and vestibular parameters including saccadic eye movements (SEM), pursuit eye movements (PEM), saccadic intrusions (SI), gaze-holding in primary gaze (spontaneous nystagmus (SN) and at eccentric gaze (gaze-evoked nystagmus (GEN) including rebound nystagmus (RBN) when returning to straight-ahead gaze after prolonged eccentric-gaze holding), angular vestibulo-ocular reflex (aVOR) testing, and otolith testing. For these parameters, we calculated the frequency of presentation by grading the percentage of abnormal findings in studies reporting on either frequency values or single patients’ data with symbols (from “Ø“ (not present) to “+++”, with distinction between rarely present (=/+, i.e. in < 20%), sometimes present (+, i.e. in 20–40%), present in a significant fraction (++, i.e. in 40–70%), and frequently present (+++, i.e. in > 70%)). Similarly, the level of abnormality was determined by calculating the difference between the mean value of the patient group and the corresponding control value. The degree of abnormality was then expressed by a symbol (from “o” (normal) to “↑↑↑/↓↓↓”), with distinction between being reduced/increased mildly (↑/↓, i.e., by < 25%), moderately (↑↑/↓↓, i.e., by 25–50%), and strongly (↑↑↑/↓↓↓, i.e., by > 50%). Details of these symbols are provided also in the legends of Tables 3 and 4. In those studies that reported individual patient data, averages (± 1SD) were calculated to allow comparison with other studies that reported group means only. Single patient’s values were collected for meta-analyses. However, this was only possible for aVOR-responses due to differences or uncertainties regarding measurement methods in all other ocular motor/vestibular domains.

Additionally, we extracted information on correlations between ocular motor/vestibular parameters and other variables such as disease severity expressed by clinical scores, age at symptom-onset, or CAG repeat-length. Spearman or Pearson correlation coefficients were classified as strong (*r* ≥ 0.7), moderate (0.4 ≤ *r* < 0.7), or weak (0.1 ≤ *r* < 0.4), respectively [39].

## Results

### Overview of Studies, Experimental Setups and Parameters

Our literature search yielded 43 unique citations (see PRISMA flow chart – Fig. 1). Six of those studies were excluded after title and abstract screening. One report could not be retrieved as the results had not yet been published [40]. Having reviewed the full text manuscripts of the remaining 36 studies, 11 citations were excluded. Another 5 studies were identified by a manual search of the reference lists of the selected 25 manuscripts. All of those 5 studies were included based on the subsequent full-text review. Thus, a total of thirty studies reporting on genetically confirmed SCA3-patients were included, with study quality being high in 6 studies, moderate in 8 studies and low in 16 studies (see Table 1). Five studies also provided data on pre-symptomatic carriers [27, 28, 31, 41, 42]. Of these, 2 included the same patient cohort that was divided into 14 subjects classified as pre-symptomatic carriers near ataxia onset (PAN) and 24 as far from ataxia onset (PAFF), with the cutoff at 4 years from the predicted age of ataxia onset (for distinguishing PAFF and PAN subcategories) based on the CAG repeat length (according to [43]) in one cohort [27, 28]. For the 3 pre-symptomatic carriers in [42], data was merged with the symptomatic patients.

**Table 1:** Study design and patient population

	Studies (*n*)	Patients (*n*)
**Study location**		
Monocentric	30 [27–38, 41, 42, 44–57, 79, 80]	432
Multicentric	0	0
**Study type**		
Case series	5 [30, 46–48, 52]	50
Case-control study	22 [27, 29, 32–38, 41, 42, 44, 45, 49, 51, 53–57, 79, 80]	305 *
Longitudinal study	2 [28, 31]	76 †
Single case report	1 [50]	1
**Data collection over time**		
Prospective	29 [27–38, 41, 42, 44–47, 49–57, 79, 80]	426
Retrospective	1 [48]	6
**Gender**		
Male patients included	20 [27, 29, 31–36, 38, 41, 42, 44, 45, 47, 50, 54, 55, 57]	155
Female patients included	20 [27, 29, 31–36, 38, 41, 42, 44, 45, 47, 54–57]	217
Gender not reported	10 [28, 30, 46, 48, 49, 51–53, 79, 80]	60
**Patient cohort**		
Pre-symptomatic carriers	5 [27, 28, 31, 41, 42]	64
Symptomatic patients	30 [27–38, 41, 42, 44–57, 79, 80]	368
**Overall study quality**		
High	6 [29, 33, 41, 42, 44, 55]	154 ‡
Moderate	8 [31, 32, 35–38, 45, 46]	126
Low	16 [27–30, 34, 47–53, 56, 57, 79, 80]	162
**EM data collection**		
Electro-oculography (EOG)	6 [30, 46, 49, 51, 56, 80]	33
EOG and search coils	1 [47]	3
Synoptophore	1 [57]	8
No EM data collection	1 [44]	14
Scleral search coils	4 [29, 33, 34, 53]	18 ||
Video-oculography (incl. Infrared)	15 [27, 28, 31, 32, 37, 38, 41, 42, 45, 48, 50, 52, 54, 55, 79] §	327
VOG and search coils	2 [35, 36]	29
**Plane of EM recording**		
Horizontal only	11 [29, 30, 33, 48, 49, 51, 53, 55, 56, 79, 80]	86
Horizontal and vertical	17 [27, 28, 31, 32, 34–38, 41, 42, 45–47, 50, 52, 54]	324
**Number of eyes recorded**		
Monocular recordings	13 [27–29, 31–36, 45, 53–55]	229
Binocular recordings	11 [37, 38, 41, 42, 44, 50, 52, 56, 57, 79, 80]	155
Not reported	6 [30, 46–49, 51]	48
All	30 [27–38, 41, 42, 44–57, 79, 80]	432

EM eye movement, EOG electro-oculography, SSC scleral search coils, VOG video-oculography

* In one study, 30 out of 73 patients had one or several follow-up visits and thus were assigned to the longitudinal study category [27]

† In one study, 22 out of 30 patients had a second follow-up visit [28]

‡ Two studies included the same patient cohort [29, 33], but were assigned to different study qualities. These 10 patients were therefore included both in the high and low overall study quality

§ In one study with 11 patients, video-oculography was recorded with a smartphone camera (iPhone 8 + and iPad Pro 12.9-inch, 2nd gen), and thus no infrared was used [79]

|| Due to the same patient cohort examined in two studies [29, 33], patients were only counted once (*n* = 10)

Video-oculography (VOG) was used in 17 studies. Other devices included electro-oculography (EOG; *n* = 7), scleral search coils (SSC; *n* = 5) and a synoptophore test device (*n* = 1). One study did not report on eye movements but on cervical and ocular vestibular-evoked myogenic-potentials (cVEMP, oVEMP) [44]. Table 2 provides an overview of the recorded ocular motor and vestibular parameters and Tables 3 and 4 summarize the frequency and level of abnormality of both ocular motor (Table 3) and vestibular (Table 4) domains tested.

**Table 2:** Recorded ocular motor and vestibular parameters

	Studies (*n*)				Patients (*n*)			
	Horizontal EM only	Vertical EM only	Vertical and horizontal EM	total	Horizontal EM only	Vertical EM only	Vertical and horizontal EM	total
**Gaze holding**								
SN in primary gaze position	4 [28, 46, 48, 80]	0	5 [27, 37, 38, 42, 47]	9 [27, 28, 37, 38, 42, 46–48, 80]	54 *	0	131	155 †
Gaze-evoked nystagmus	5 [28, 42, 48, 50, 80]	0	6 [27, 37, 38, 41, 46, 47]	11 [27, 28, 37, 38, 41, 42, 46–48, 50, 80]	86 *	0	156	212 †
Rebound nystagmus	1 [80]	0	2 [46, 47]	3 [46, 47, 80]	1	0	10	11
**Triggered nystagmus**								
Head shaking nystagmus	0	0	2 [37, 38]	2 [37, 38]	0	0	17	17
Optokinetic nystagmus	3 [42, 46, 80]		0	3 [42, 46, 80]	45	0	0	45
Positional nystagmus	0	0	2 [37, 38]	2 [37, 38]	0	0	17	17
Pursuit eye movements	3 [46, 79, 80]	2 [28, 48]	5 [27, 34, 37, 41, 47]	9 [27, 28, 34, 37, 41, 46–48, 79, 80]	19	68 *	144	201 †
**Saccadic eye movements**								
Visually guided saccades	7 [29, 38, 46, 48, 49, 51, 80]	2 [27, 28]	6 [37, 41, 42, 45, 47, 50]	15 [27–29, 37, 38, 41, 42, 45–51, 80]	61	73	110	244 †
Saccadic intrusions	3 [46, 48, 80]	0	7 [37, 38, 41, 45, 47, 50, 52]	10 [37, 38, 41, 45–48, 50, 52, 80]	24	0	89	113
**aVOR**								
Caloric irrigation	2 [30, 56]	0	0	2 [30, 56]	9	0	0	9
Decay TC	1 [80]	0	0	1 [80]	1	0	0	1
Rotational	3 [46, 53, 80]	0	0	3 [46, 53, 80]	10	0	0	10
Translational	1 [53]	0	0	1 [53]	2	0	0	2
Vision-enhanced	1 [46]	0	1 [34]	2 [34, 46]	7	0	6	13
aVOR high frequency	6 [27, 28, 33, 34, 42, 55]	0	6 [31, 32, 35–37, 54]	12 [27, 28, 31–37, 42, 54, 55]	162 †	0	92	254 †
aVOR-suppression	2 [46, 80]	0	1 [34]	3 [34, 46, 80]	8	0	5	13
**Total EM recordings**	11	3 ‡	17	28	86	111 ‡	324	410

EM eye movement, aVOR angular vestibulo-ocular reflex, OKN optokinetic nystagmus, SN spontaneous nystagmus, TC time constant

* Including 30 patients from [28] that were also part of the patient cohort from [27], where horizontal and vertical eye movements were recorded

† Patients (*n* = 30) from [28] were not included here, because they were part of the 73 patients included from [27]

‡ Note that all patients from the studies in this column appear in the “horizontal EM only” or “vertical and horizontal EM” column as well, therefore neither the studies nor the patients were included in the “total” column

**Table 3:** Overview of ocular motor findings – level of abnormality and frequency of presentation

	Level of abnormality *	*n*	Frequency of presentation	*n*
**Pursuit eye movements** †				
PEM gain hor	↓ – ↓↓ [34, 41, 42, 46]	107	++ – +++ [34,37,46] ‡	19
PEM gain ver	↓ [27, 41] §	129		0
Saccadic pursuit		0	=/+ [48] ||, +++ [37,47]	26
**Saccadic eye movements**				
VGS hor – increased latency	o – ↑ [42,45,46]	51	=/+ – ++ [45,46,48]	29
VGS ver – increased latency	o [41]	56		0
VGS hor – velocity #	↓ – ↑ [41,42,45,46,49]	119	Ø – + [29,37,45–51]	65
VGS ver – decreased velocity	↓↓ [41]	56		0
VGS hor – dysmetric		0	+ – +++ [29,37,38,45–48]	59
VGS hor – hypermetric **	o – ↑↑ [29,41, 46]	73	=/+ – +++ [29, 37, 45–47]	32
VGS hor – hypometric	o [41]	56	Ø – ++ [29, 37, 45–47]	32
AS – error rate	↑↑↑ [41]	56		0
VVS slope	↑↑ [27] ††	73		0
RVS slope	↑ – ↑↑ [27] ††	73		0
**Saccadic intrusions**				
Square-wave jerks	↑↑↑ [41, 45]	62	++ – +++ [37,38,41,45–47] §§	45
Ocular flutter		0	Ø – =/+ [38,46]	18
Micro-opsoclonus		0	+++ [52]	6
Macro-saccadic oscillations		0	=/+ [37]	6
**Spontaneous nystagmus**				
Downbeat nystagmus		0	Ø – =/+ [38, 46, 47]	21
Horizontal nystagmus		0	Ø – =/+ [37, 38, 48]	33
Eccentric gaze holding				
Gaze-evoked nystagmus hor - SPV	↑↑↑ [27, 42] ‡‡	111	+++ [37, 38, 42, 46–48, 50]	81
Rebound nystagmus		0	+ – +++ [46, 47]	10
**Triggered nystagmus**				
Head-shaking nystagmus hor		0	Ø – + [37, 38]	17
Positional nystagmus (dynamic testing)		0	Ø – =/+ [37, 38] ||||	17
OKN impairment - gain	↓↓ - ↓↓↓ [42,46]	44	+++ [46]	6
**Symbol legend:**				
	Level of abnormality: o = normal ↑/ ↓ = reduced/increased by <25% (mildly) ↑↑/↓↓ = reduced/increased by 25–50% (moderately) ↑↑↑/↓↓↓ = reduced/increased by > 50% (strongly)		Frequency of presentation: Ø = not present =/+ = fraction <20% (rarely) + = fraction 20–40% (sometimes) ++ = fraction 40–70% (in a significant fraction) +++ = fraction > 70% (frequently)	

*AS* anti-saccades; *hor* horizontal; *OKN* optokinetic nystagmus; *PEM* pursuit eye movement; *RVS* reflexive vertical saccades; *SPV* slow phase velocity; *ver* vertical; *VGS* visually guided saccades; *VVS* volitional vertical saccades

*Included studies provided the degree of change in a quantitative way or stated that results were within normal limits.

†Comparison difficult due to differences in measurement methods (amplitude, velocity, frequency)

‡Missing information on plane of measurement, presumably horizontal, but not further specified [34]

§In one study, smooth pursuit gain was slightly increased for pre-symptomatic carriers and decreased for ataxic carriers [27]

|| In one study, smooth pursuit assessment didn't cover all patients and therefore saccadic pursuit might have been under-detected [48]

¶No mean control value for [46], therefore comparison with control value from [45]

# Comparison difficult due to different measurement methods (amplitudes 5–30°, peak and mean velocities)

**Level of abnormality refers to hypermetria in %. One study assessed dynamic overshoot [29]

††In this study, median values were compared because no mean values were provided. Pre-symptomatic carriers far from ataxia onset showed mild changes (↑) for both VVS and RVS slopes, whereas pre-symptomatic carriers near ataxia onset showed moderately increased VVS slopes (↑↑) and mildly increased RVS slopes (↑). Ataxic carriers showed both moderately increased VVS and RVS slopes (↑↑) [27]

‡‡No reference value for [42], therefore comparison with control value from [27]

§§In [41] only frequency of presentation for pre-symptomatic carriers was reported

|||| Static testing demonstrated higher percentage (++) of positional nystagmus in [38]

**Table 4:** Overview of vestibular findings – level of abnormality and frequency of presentation

	Level of abnormality	*n*	Frequency of presentation	*n*
**Low-frequency aVOR**				
Gain reduction	↓ – ↓↓↓ [46, 53]	9	++ [46, 53]	9
aVOR suppression				
Impaired (gain)	o – ↑ [34, 46]	12	++ [34, 46]	12
High-frequency aVOR (qHIT) *				
HC gain reduction	↓↓ [27, 28, 31–37, 42, 54, 55]	254	+++ [31–35, 37, 54, 55] †	162
AC gain reduction	↓↓ [32, 35–37, 54]	77	+++ [37, 54]	27
PC gain reduction	↓↓ [32, 35–37, 54]	77	++ – +++ [37, 54]	27
Catch-up saccades during vHIT	↑↑↑ [55] ||	15	+++ [37, 55] ¶	21
SHIMP HC gain reduction	↓↓ [54]	21		0
**VVOR**				
Gain reduction	↓↓ [34]	6	+++ [34]	6
Caloric irrigation				
Decreased or absent response		0	++ – +++ [30, 56]	9
**cVEMP**				
Absent reflex or prolonged latency either left or right	o [35, 36, 54] - ↑↑↑ [44]	64	=/+ – +++ [35, 36, 44, 54, 56] #	66
**oVEMP**				
Absent reflex either left or right		0	+++ [44] **	14
**Symbol legend:**				
	Level of abnormality: o = normal ↑/ ↓ = reduced/increased by < 25% (mildly) ↑↑/↓↓ = reduced/increased by 25–50% (moderately) ↑↑↑/↓↓↓ = reduced/increased by > 50% (strongly)		Frequency of presentation: Ø = not present =/+ = fraction < 20% (rarely) + = fraction 20–40% (sometimes) ++ = fraction 40–70% (in a significant fraction) +++ = fraction > 70% (frequently)	

*AC* anterior canal; *aVOR* angular vestibulo-ocular reflex; *cVEMP* cervical vestibular evoked myogenic potential; *HC* horizontal canal; *n* number of patients undergone testing; *oVEMP* ocular vestibular evoked myogenic potential; *PC* posterior canal; *PV* peak velocity; *SCC* semicircular canal; *SHIMP* suppression head impulse test protocol; *qHIT* quantitative head-impulse testing; *VVOR* visually-enhanced VOR

*In two studies, regression slopes of VOR gain (VORr) was calculated [27, 55]

†In one study frequency of presentation was lower (++) in pre-symptomatic carriers [31]

‡Only mildly reduced for left PC in one study [35]

|| Level of abnormality refers to increased eye PV compared to controls

¶Overt saccades more frequent than covert saccades in both studies [37,55]

# Absolute percentage of patients with absent response or prolonged latency (percentage of controls with absent response or prolonged latency between 10–29%)

**Absolute percentage of patients with absent response (percentage of controls with absent response 0%)

Correlations between oculomotor/vestibular changes and other parameters were retrieved from 9 studies [27, 31–33, 37, 41, 42, 44, 45], of which 2 studies included longitudinal data [27, 31]. For more data see Appendix 3.

### Ocular Motor Findings

#### Pursuit Eye Movements (PEM)

**Fig. 2:**
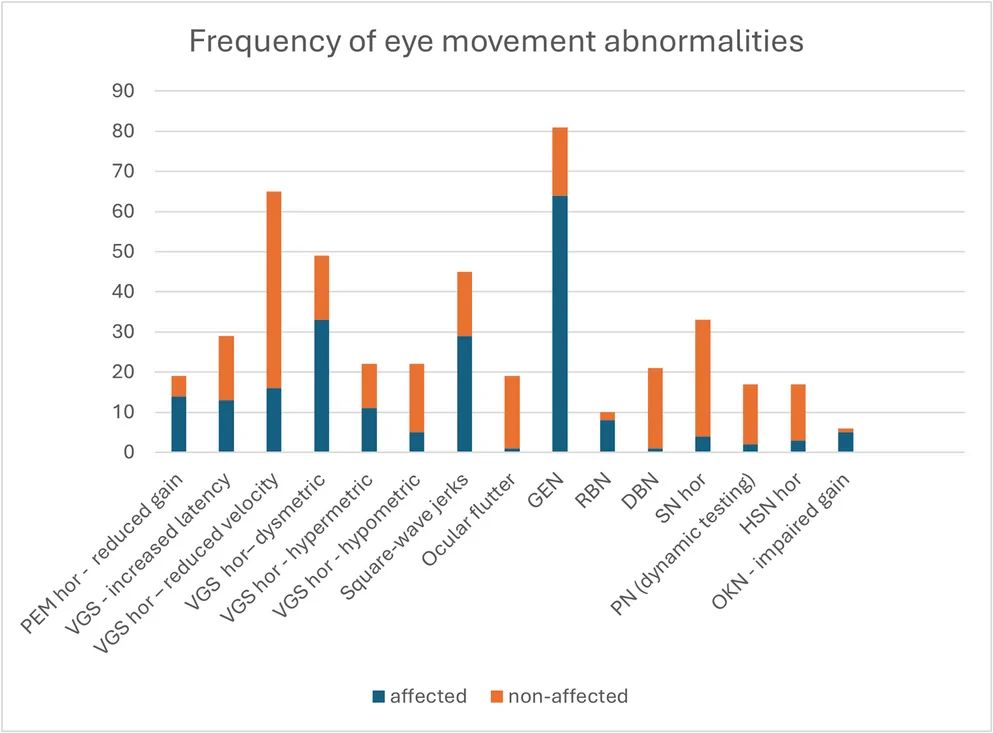
**Frequency of eye movement abnormalities. ** Frequency of eye movement abnormalities for all SCA3 patients included. For each parameter the number of affected individuals (in blue) and non-affected individuals (in orange) is shown. *Abbreviations* DBN = downbeat nystagmus; hor = horizontal; HSN = head-shaking nystagmus; GEN = gaze-evoked nystagmus; PEM = pursuit eye movement; PN = positional nystagmus; OKN = optokinetic nystagmus; RBN = rebound nystagmus; SN = spontaneous nystagmus; VGS = visually guided saccades

PEM gain was recorded in 227 subjects from 8 studies [27, 34, 37, 41, 42, 46–48]. In the studies providing single subject data, horizontal PEM gain was impaired in most patients (14/19, 74%) [34, 37, 46] (see Fig. 2). On a group level, horizontal PEM gain was mildly to moderately reduced in all studies when compared to controls [34, 41, 42, 46]. Considering vertical PEM gain, a mild reduction for symptomatic patients was reported compared to controls in one study [27], while no significant reductions were noted for pre-symptomatic carriers. In contrast, another study described lower vertical PEM gain in pre-symptomatic carriers compared to healthy controls [41]. PEM were rated as “saccadic” in 10/25 (40%) SCA3 patients. In 2 studies saccadic PEM were present in all patients (*n* = 9) [37, 47], whereas this was the case in only 1/16 (6%) patients in a third study, probably due to not having performed PEM assessment in all patients [48]. Another study reporting on 56 patients including 12 pre-symptomatic carriers found broken-up horizontal PEM to be a key clinical feature of SCA3 without reporting on its frequency [41].

While the *horizontal* PEM gain demonstrated significant correlations with disease duration [41] and the SARA (Scale for the Assessment and Rating of Ataxia) score [41, 42], this was not the case for the *vertical* PEM gain [27]. However, the same study reported weak negative correlations between vertical PEM-gain and ICARS (International Cooperative Ataxia Rating Scale) as well as CCFS (Composite Cerebellar Function Score) and weak positive correlations with SCAFI (Spinocerebellar Ataxia Functional Index) and several disease duration metrics. Specifically, in this study, a weak positive correlation between vertical PEM gain and estimated time to ataxia onset in pre-symptomatic carriers and disease duration in symptomatic patients [27].

## Saccadic Eye Movements (SEM)

Most studies reporting on saccades to target referred to visually-guided saccades (VGS) in the horizontal plane [29, 37, 41, 42, 45–51], except for two studies that reported on self-paced volitional vertical saccades (VVS) and reflexive vertical saccades (RVS) [27] or anti-saccades (AS) [41].

### Saccade Latency

Latency (or delay) of horizontal VGS was either not [46] or mildly (< 12%) [42, 45] increased compared to controls. Percentages of SCA3-patients with prolonged horizontal VGS-latency ranged from 14% (1/7) [46] to 63% (10/16) [48]. The only study reporting on VGS latency in the vertical plane did not observe any significant abnormalities [41].

Correlations between SARA and saccade latency were strong for symptomatic patients in one study [45], but weak for both pre-symptomatic carriers and symptomatic patients in another study [41]. Additionally, saccade latency demonstrated a weak correlation with disease duration [41].

### Saccade Accuracy

Dysmetric saccades were observed in 33/49 patients (67%) [29, 37, 38, 45–48]. Where specified, saccades were more often reported to be hypermetric (11/22, 50%) than hypometric (5/22, 23%) [37, 45–47], while both horizontal and upward saccade accuracy were normal on average in pre-symptomatic carriers [41]. One study reported on dynamic overshoot, which is an eye movement surpassing the desired end position that is corrected without a break (conversely to hypermetric saccades, where the corrective eye movements are initiated after a short pause and thus a new motor process) [29]. While 0/10 controls had dynamic overshoot greater than 2%, this was observed in 7/10 (70%) SCA3-patients.

The AS error-rate was increased in pre-symptomatic carriers (36.0 ± 24.1%) compared to controls (19.2 ± 14.0%) and further increased in SCA3-patients (66.8 ± 22.9%) in one study [41]. Horizontal and vertical saccade accuracy showed moderate inverse correlations with the SARA score in the same study [41]. Similarly, the AS error-rate demonstrated moderate correlations with the SARA score and weak correlations with both the CAG repeat-length and disease duration [41]. Horizontal and vertical (upward) saccadic accuracy revealed a weak and moderate negative correlation with the latter [41].

### Saccade Velocity

Horizontal VGS peak velocity or the ratio between measured and expected peak velocity was mildly reduced in 7/29 patients (24%) [29, 46, 49]. When focusing on VGS mean velocity, 3/11 patients (27%) demonstrated impairment [45, 50, 51]. At a study level, VGS velocities did not differ significantly between patients and controls in 2 studies [41, 46] and in another 2 studies velocity was mildly reduced [45, 49]. Two studies compared adductive and abductive horizontal VGS. One of these studies reported a greater than normal velocity for adducting saccades whilst the velocity of abducting saccades was in the normal range [42]. Similarly, in a single case report, a SCA3 patient demonstrated lower mean VGS velocities for abduction compared to adduction without providing control values [50].

Three studies described that slowing of vertical saccades was more pronounced than for horizontal saccades [41, 47, 50]. This was restricted to upward saccades in both SCA3-patients and pre-symptomatic carriers in one study [41]. The same study revealed a weak inverse correlation between (upward) saccades peak velocity and disease duration [41].

VVS slope and RVS slope (peak duration vs. amplitude) were calculated and reportedly augmented by 45.4% for VVS, and 43.2% for RVS, respectively, between symptomatic patients and controls in one study [27]. Additionally, RVS and VVS differed significantly between pre-symptomatic carriers near ataxia onset and controls and sometimes deteriorated years before the predicted age of symptom-onset [27]. Noteworthy, all correlations of RVS and VVS with clinical scores other than the correlation of VVS with SARA were significant (weak to moderate) and both correlated moderately with disease duration and estimated time to symptom onset (in pre-symptomatic carriers), as well as Z-scores and time to symptom-onset in pre-symptomatic carriers [27] (see Appendix 3).

### Saccadic Intrusions (SI)

Saccadic intrusions were present in 38/62 patients (61%) [37, 38, 41, 45–48, 50, 52], being studied either at eccentric gaze or gaze straight-ahead. The most frequently reported saccadic intrusion were square-wave jerks (SWJ). Of note, details on measurement methods in these studies were frequently omitted. SWJ-frequency was reported in a single study, ranging from 8 to 50 SWJ over 60 s intervals in single patients (control group: mean 1.2 SWJ/60 s) [45]. In another study, increased frequency and amplitude of gaze-evoked SWJ were present in all but one (11/12) pre-symptomatic carriers [41]. In symptomatic patients the SWJ frequency and amplitude showed a weak correlation with the SARA score [41]. Additionally, gaze-evoked eye movements (i.e., gaze-evoked nystagmus or SWJ during eccentric gaze, GEEM) were reported more frequently present during upward gaze than during central fixation [41]. In one SCA3-patient, a pattern of monocular/disconjugate alternating type of SI was identified [50].

Other saccadic intrusions included micro-opsoclonus (5/6 patients [52]), ocular flutter (1/19 [38, 46]), macro-saccadic oscillations (1/6 [37]), square-wave oscillations (3/11 [38]) and single saccadic pulses (1/1 [50]).

### Spontaneous and Gaze-Evoked Nystagmus

Eccentric gaze holding was abnormal in 79% (65/82) of patients examined (pooled results of seven studies) [37, 38, 42, 46–48, 50]. The prevalence of GEN at 30° horizontal gaze deviation in two studies was 31/39 (79%) [42, 50]. In another two studies which measured both horizontal and vertical gaze deviation at 30°, it was seen in 16/18 patients (89%) [38, 46]. The frequency of rebound nystagmus (RBN) varied between 33% (1/3) [47] and 100% (7/7) [46], albeit sample sizes were small.

Spontaneous nystagmus (SN) was reportedly present in a number of studies and with variable frequency between 0% [37, 46, 47] and 18–19% [38, 48]. Of 21 patients examined, spontaneous downbeat nystagmus (DBN) in primary gaze was identified in 2 patients (10%) [35, 46, 47]. SN in the horizontal plane was observed in 4/30 patients (13%) [37, 38, 48].

Correlations between SN slow-phase velocity (SPV) and clinical scores were either insignificant or weak in one study [27], but for GEN SPV they reached a moderate to high level for all parameters assessed in two studies [27, 42] (for details see Appendix 3). Moreover, GEN SPV and CAG repeat-length demonstrated a weak to moderate correlation [42]. A strong correlation was found between horizontal GEEM amplitude and SARA, to a lesser extent also for GEEM frequency [41]. Both GEEM frequency and amplitude correlated moderately with disease duration [41]. SN and GEN SPV correlated with disease duration in SCA3 patients and with estimated time to symptom-onset in pre-symptomatic carriers in one study, with high correlation coefficients for GEN SPV [27].

### Triggered Nystagmus

Head-shaking nystagmus and positional nystagmus were examined in 2 studies and 17 patients [37, 38]. Neither of them was present in one study [37], whilst horizontal head-shaking nystagmus was seen in 3/11 SCA3-patients following horizontal head shaking in the other [38]. Similarly, positional DBN and horizontal nystagmus, were each observed in one patient (during the Dix-Hallpike manoeuvre) [38]. During supine-roll testing or straight-head hanging, horizontal positional nystagmus was seen in 5/11 patients [38]. Optokinetic nystagmus (OKN) gain was decreased by 63.8% [46] and 36.5% [42] in two studies, respectively. OKN SPV showed moderate inverse correlations with SARA, CAG repeat-length and disease duration [42].

### Vestibular and Vestibular-Visual Findings

#### Angular Vestibulo-Ocular Reflex (aVOR), aVOR-Suppression and Visually-Enhanced VOR (VVOR)

**Fig. 3:**
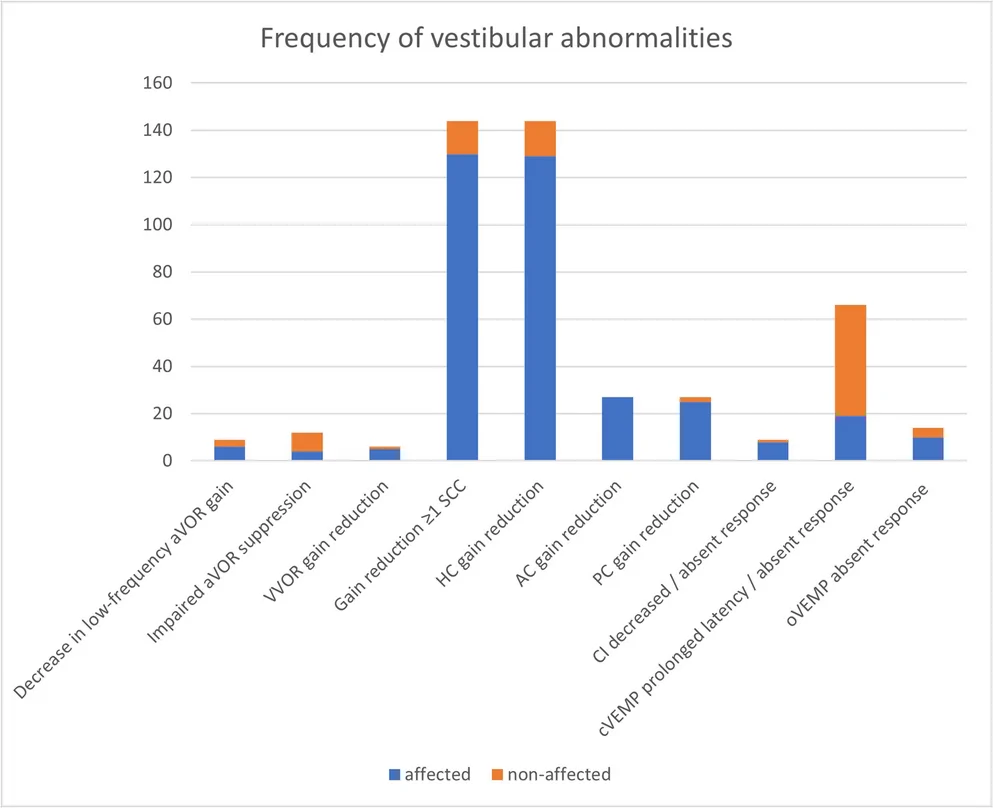
**Frequency of vestibular abnormalities. **Frequency of vestibular abnormalities for all SCA3 patients included. For each parameter the number of affected individuals (in blue) and non-affected individuals (in orange) is shown. *Abbreviations*AC= anterior canal; aVOR = angular vestibulo-ocular reflex; CI = caloric irrigation; cVEMP = cervical vestibular evoked myogenic potential; HC = horizontal canal; oVEMP = ocular vestibular evoked myogenic potential; PC = posterior canal; SCC = semicircular canal; VVOR = visually enhanced VOR

Low frequency aVOR-gains were variably reduced depending on the oscillation frequencies studied (0.05 to 1.25 Hz) [46, 53] (see Fig. 3). Mean aVOR gain at 0.05 Hz for 9 patients from these studies [36, 44] was 0.30 ± 0.17. aVOR-suppression was severely impaired in 3/7 patients (43%) in one study [46], whereas it was preserved for all patients (*n* = 5) in another study [34]. The visually-enhanced VOR was tested in one study, revealing abnormally low gains (< 0.8) in 5/6 (83%) SCA3 patients [34].

### Quantitative Head-Impulse Test (qHIT) and Caloric Irrigation

qHIT results were retrieved for 254 patients (lateral canals testing only in 162 patients) using search coils in 16 patients [33, 34] or video Head Impulse Test (vHIT) in 238 patients [27, 28, 31, 32, 35–37, 42, 54, 55]. qHIT-gains were decreased in ≥ 1 semicircular canal in 117/133 patients (88%) [31–35, 37, 54, 55]. Average of horizontal qHIT-gains (± 1SD) in symptomatic SCA3-patients in 10 studies [27, 31–37, 54, 55] ranged from 0.39 ± 0.16 [34] to 0.65 ± 0.20 [42]. The calculated regression slope of the horizontal VOR (VORr) averaged at 0.50 ± 0.30 for 15 patients in one study [55] and at 0.65 ± 0.24 for 35 patients from another study [27].

VORr/aVOR gain during the vHIT correlated with disease severity moderately to strongly in two studies [27, 32] (for details see Table 5). For vHIT, a moderate to strong correlation with SARA was confirmed in 4 studies [31, 33, 37, 42]. However, neither horizontal aVOR-gain in another study [33] nor anterior-canal gain, posterior-canal gain or anterior/posterior canal ratio correlations with SARA reached the level of significance in another study [37]. Additionally, VORr correlated with disease duration in SCA3-patients and estimated time to symptom onset in pre-symptomatic carriers [27]. While one study revealed moderate correlations between vHIT and disease duration as well as CAG repeat length [42], another study demonstrated a weak correlation for the latter comparison only [31] and in a third study both comparisons remained non-significant [37]. In one study the percentage of change in horizontal aVOR-gain negatively correlated with the percentage of change in the SARA score across 2 examinations 9–24 months apart (*r*=−0.54, *p* < 0.05) [31].

**Table 5:** Correlations: aVOR (Spearman) *

	SARA	CAG repeat length	Disease duration	Time to/time after onset †	DHI	NESSCA	ICARS	INAS count	SCAFI	CCFS
Hor high frequency aVOR (qHIT)	NS [33], moderate to strong [31] ‡ ||, [27, 37, 42] ||	NS to moderate [31] § ||, [37, 42] ||	NS to moderate [37, 42] ||	moderate § to strong [27] ||	moderate [32]	strong [27] ||	strong [27] ||	moderate [27] ||	moderate [27] §	moderate [27] § ||

aVOR angular vestibulo-ocular reflex, CCFS Composite Cerebellar Function Score, GEEM gaze-evoked eye movement, GEN gaze-evoked nystagmus, hor horizontal, ICARS International Cooperative Ataxia Rating Scale, INAS count Inventory of Non-Ataxic Signs, NESSCA Neurological Examination Score for Spinocerebellar Ataxia, OKN optokinetic nystagmus, PD peak duration, PV peak velocity, qHIT quantitative head-impulse test, RVS reflexive vertical saccades, SARA Scale for Assessment and Rating of Ataxia, SCAFI Spinocerebellar Ataxia Functional Index, SN spontaneous nystagmus, SPV slow-phase velocity, SWJ square wave jerks

* Only parameters with at least one significant correlation are shown and classified as following: strong correlations: *r* ≥ 0.7; moderate: 0.4 ≤ *r* < 0.7; weak: 0.1 ≤ *r* < 0.4

† Moderate Pearson correlation between Z-scores and time to onset in pre-symptomatic carriers and both strong for time to/time after onset of ataxic and pre-symptomatic carriers and time to/time after ataxia onset using predicted age of ataxia onset at birth

‡ Additional significant Pearson correlation analyses between horizontal aVOR gain and SARA score in 20 patients in two examinations and between change in percent of the aVOR gain and change in percent of the SARA score in 20 patients across two examinations

§ Pearson correlation analysis

|| Negative correlation

Regarding the distinction between pre-symptomatic carriers and controls, the aVOR gain during vHIT was abnormally low in a significant fraction of pre-symptomatic carriers (54%) vs. controls (0%) in one study [31]. Similarly, in comparison with controls, the VORr was significantly different in pre-symptomatic carriers near ataxia onset and symptomatic patients, but not in pre-symptomatic carriers far from ataxia onset [27].

Aside from impaired aVOR-gains, an increased prevalence of overt, and covert saccades was reported in 18/21 (86%), and 7/21 (33%) SCA3-patients, respectively, in two studies [37, 55]. Caloric irrigation in a small sample of patients demonstrated frequently decreased or absent responses (8/9, 88.9%) [30, 56].

### Vestibular Evoked Myogenic Potentials (VEMPs)

Frequencies of absent cervical vestibular evoked myogenic potentials (cVEMPs) ranged from 10% [54] to 66% [56] (mean = 32.4%) in 5 studies [35, 36, 44, 54, 56], with responses missing bilaterally in 7/47, and unilaterally in 6/47 patients [36, 44, 54, 56]. In these studies, percentage of controls with altered response ranged from 0 to 29% [35, 36, 44, 54, 56]. Peak-to-peak amplitudes were normal for cVEMPs in 2 studies [35, 36] and decreased for both cVEMPs and oVEMPs in one study [44]. Both cVEMPS and oVEMPs were assessed in a single study only [44], with missing oVEMP responses in 10/14 patients (71%) vs. 0/20 controls (0%). In this study, abnormal oVEMPs or cVEMPs were seen in 13/14 (93%) patients, whose cVEMP and oVEMP responses were asymmetric in 9/13 (69%), meaning 4/13 showed absent oVEMPs but present cVEMPs, another 2/13 had missing cVEMPs but normal oVEMPs, and 3/13 patients had unilateral prolonged cVEMP latencies [44].

### Vergence Disorders

Vergence rates were assessed by use of a synoptophore test in a single study with 8 patients. While five patients had isolated divergence (*n* = 3) or convergence (*n* = 2) disorders, three patients were found to have both decreased convergence and divergence rates [57]. Prism tests revealed greater esodeviation in lateral gaze at near (24/38, 63%) and at distance (29/38, 76%) in another study [42].

## Discussion

This systematic review focuses on quantitative ocular motor and vestibular testing in SCA3 patients with a molecular diagnosis, in order to identify potential biomarkers suitable for early disease detection, monitoring disease progression and treatment response. The most frequently demonstrated ocular motor/vestibular abnormalities identified in our review were angular vestibulo-ocular reflex (aVOR) gain reductions as measured by quantitative head-impulse testing (88%), impaired eccentric gaze holding (horizontal gaze-evoked nystagmus [GEN] in 79%, combined horizontal and vertical GEN in 89%), reduced horizontal pursuit gain (74%), saccadic dysmetria (67%), and fixation instabilities (square-wave jerks in 64%). Other changes such as visually enhanced VOR gain reductions and impaired response on caloric irrigation, were commonly identified, but studied only in small patient cohorts, limiting the impact of these findings [58–60]. Contrary to another review that depicted strabismus and vergence disorders as typical features of SCA3 [58], we failed to find clear affirmations, probably due to lack of quantitative data as of 6 studies reporting on it [42, 47, 50, 52, 53, 57], only two studies provided quantitative results [42, 57].

### Saccade Metrics and the Impact of the Direction of saccades – Abduction vs. Adduction

Although saccadic abnormalities such as slowing or dysmetria were frequently observed, changes were often mild, possibly due to averaging of saccades resulting from adducting and abducting eye movements [42]. Indeed, correlations including abducting saccades were higher compared to those including adducting saccades and only the amplitudes of abducting saccades were decreased compared to those retrieved in healthy controls [42]. Similarly, velocities of abducting saccades were decreased compared to adducting saccades [42, 50] and single saccadic pulses (i.e., saccadic intrusions) were highly asymmetric [50]. According to [42], the reason may be abducens nucleus involvement resulting in abduction palsy. The faster than normal adducting saccades on the other hand, might reflect fastigial nucleus impairment or an adaptive reorganization of the saccadic network in response to chronic strabismus to compensate for abduction deficits [42]. Further research on these disconjugate patters of saccades and saccadic intrusions in SCA3 is needed.

Noteworthy, saccadic accuracy was normal in pre-symptomatic carriers, while both hypermetric and hypometric saccades were frequently observed in symptomatic patients and correlated with disease duration [41]. This is supported by the fact that of all studies reporting on the frequency of dysmetric saccades, it was lowest (38%) in the patient cohort with the shortest disease duration [48]. It has been proposed that inaccurate or slow saccades in SCA3 might emerge from deficits in the saccade velocity feedback resulting from impairment of medial cerebellar lobules V-VII, leading either to low gain without overshoot or to high gain with overshoot [29].

A strongly increased anti-saccade error-rate for both SCA3 patients and pre-symptomatic carriers was observed in a single study [41]. While no information on cognitive function was available in this study, these findings likely reflect executive dysfunction as part of the cerebellar cognitive affective syndrome, as previously reported in SCA3 patients by others [61, 62] and also summarized in a recent systematic review [63]. Noteworthy, another parameter that has been linked to higher cognitive function, i.e., saccade latency, showed little or no changes in the studies included in our review.

### Prominent Impairment of Vestibulo-Ocular Reflexes and Mixed Results for Otolith-Mediated Reflexes

The most striking abnormality identified were aVOR-gain changes. Impaired responses were observed frequently (affecting often all canals) and consistently across different testing methods including caloric irrigation, quantitative HIT, and VVOR. Moreover, the area under the curve of aVOR-gains during quantitative HIT for discrimination between SCA3 patients and healthy controls were high (0.997 [27], 0.993 [54]). Therefore, aVOR-gain seems to be an excellent classifier to differentiate between SCA3 patients and controls and its impairment reflects a distinctive feature that could be a potential neurophysiological biomarker of SCA3, as emphasized by [54].

In contrast, vestibular-evoked myogenic potential (VEMP) testing yielded less consistent results. With cervical VEMP (cVEMP) responses being normal in most SCA3-patients [35, 36, 54], it has been proposed that such discrepancy between impaired aVOR-gain and normal cVEMPs could be explained by selective, mostly medial and superior vestibular nuclei degeneration [54]. Contrary to this, cVEMPs were abnormal in most patients in two studies with younger SCA3-patients [44, 56]. Specifically, cVEMP-responses were absent in a higher fraction (right side = 43%; left side = 36% [44]) compared to the other studies (10–20% [35, 36, 54]). Overall, this study found a high proportion of abnormal VEMP-responses (93%), albeit more often for ocular VEMPs than for cVEMPs [44]. The authors of another study proposed a degeneration of the vestibular nuclei or the peripheral vestibular system, reflected by abnormal VEMP and caloric test responses [56].

#### Stronger Impairment of Vertical than Horizontal Eye Movements

For several ocular motor parameters, we observed more pronounced impairment for the vertical plane compared to the horizontal plane. This was true for gaze-evoked eye movements (including SWJ and GEN), which were more frequent during upward gaze than during central fixation [41] and for GEN-prevalence, which was slightly higher in studies where it was searched in both horizontal and vertical gaze deviation [38, 46, 47]. Yet, the reason for the increase remains unclear. Furthermore, vertical pursuit gain was decreased in pre-symptomatic carriers and symptomatic patients, while horizontal pursuit gain was preserved [41]. Similarly, slowing of visually-guided saccades was seen more often when tested in the vertical plane [41, 47, 50]. Extensive midbrain atrophy on MRI [41] and involvement of the basal ganglia [46] have been proposed as potential explanations for vertical plane eye movements being more strongly affected in SCA3 patients. Early affection of the rostral interstitial nucleus of the medial longitudinal fasciculus and the interstitial nucleus of Cajal has been brought up as a potential explanation by others [41, 64]. Noteworthy, only a subset of studies included vertical eye movements in their analyses, which may explain also why we failed to find differences between the planes for other parameters.

#### Anatomical Correlates of Ocular Motor/Vestibular Deficits Observed in SCA3

While the most commonly affected ocular motor and vestibular parameters in SCA3, aVOR and eccentric gaze holding, may be due to dysfunction of the medial vestibular nucleus and the nucleus prepositus hypoglossi [30, 33, 37], we were unable to identify any studies reporting on structural alterations of these specific regions. However, impairment of associated structures, including the medial longitudinal fascicle, the central tegmental tract and the inferior cerebellar peduncle have been described [65]. Overall, imaging confirmed widespread neuronal loss and decreased functional connectivity [66]. One study employing magnetic resonance imaging (MRI) demonstrated atrophy of especially the anterior cerebellar regions in pre-symptomatic carriers, spreading to the rest of the cerebellum as disease progressed [67]. Thus, even in early stages, morphological imaging shows impairment of structures relevant for eye movements, which may explain the early alterations of ocular motor parameters, underscoring their importance as biomarkers. All the more so considering the fact that atrophy is visible in other areas such as the spinal cord at early stages, but without corresponding symptoms [68].

#### Proposed Ocular Motor/Vestibular Paradigms in SCA3 Patients

Here we set out to identify a core set of ocular motor and vestibular paradigms for use in SCA3 diagnosis. This set of recommendations is based on the most frequently observed and discernibly abnormal eye movements identified in the literature (see Table 6), emphasizing the co-occurrence of certain abnormalities and the absence or others.

**Table 6:** SCA3 ocular motor/vestibular “fingerprint”

Domain	Key ocular motor/vestibular changes
Gaze-holding (eccentric gaze)	• Frequent GEN [37, 38, 42, 46–48, 50] • RBN [46, 47] in a significant fraction
Triggered nystagmus	• Frequently [46] moderately to severely [42, 46] impaired OKN gain • Frequent horizontal positional nystagmus during supine-roll testing or straight-head hanging [38]
Pursuit eye movements	• Horizontal PEM-gain frequently, [34, 37, 46] but mildly impaired [34, 41, 42, 46] • PEM mostly saccadic [37, 47, 48]
Saccadic eye movements	
Saccadic intrusions	• SWJ with increased amplitude in a significant fraction [37, 41, 45–47]
Anti-saccades	• Strongly increased AS error-rate [41]
Visually guided saccades	• Velocity [29, 37, 41, 42, 45–49, 51] and latency [42, 45, 46, 48] of horizontal VGS without major changes, but moderately reduced velocity for vertical VGS [41] • Slightly [29, 41, 46] dysmetric (hypometric or hypermetric) horizontal saccades in a significant fraction [29, 37, 38, 45–48]
Angular vestibulo-ocular reflex (aVOR)	
Quantitative HIT	• Frequent gain reduction [31–35, 37, 54, 55] of moderate extent [27, 28, 31–37, 42, 54, 55] • Frequent catch-up (overt > covert) saccades during quantitative HIT [37, 55]
Caloric irrigation	• Response frequently decreased or absent [30, 56]
VVOR	• Frequent gain reductions to a moderate extent [34]
Vestibular-evoked myogenic potentials (VEMPs)	
oVEMP	• Frequently absent responses, but no latency abnormalities if responses are present [44]
cVEMP	• Sometimes absent responses or with prolonged latency [35, 36, 44, 54, 56]

AS anti-saccades, aVOR angular vestibulo-ocular reflex, cVEMP cervical vestibular evoked myogenic potential, DBN downbeat nystagmus, GEN gaze-evoked nystagmus, HIT head-impulse test, OF ocular flutter, oVEMP ocular vestibular evoked myogenic potential, RVS reflexive vertical saccade, SCA3 spinocerebellar ataxia type 3, SI saccadic intrusions, SWJ square-wave jerks, VVOR visual enhanced VOR, VVS volitional vertical saccade

In summary, the presence of increased saccadic intrusions (occasionally asymmetric between eyes or even apparently monocular with a prominent adductive component), moderate reduction of high-frequency aVOR-gains on head-impulse testing, symptomatic esotropia at far which mildly increases in lateral gaze, and almost always no spontaneous and/or relevant positional nystagmus point to SCA3. However, the additional presence of GEN, saccadic pursuit, low gain OKN, and mild slowing of upward saccades are nonspecific. Thus, potential overlaps with other cerebellar ataxias must be considered. In reality this is further bolstered by considering the broader clinical presentation of the individual patient. For instance, neither saccadic dysmetria nor reduced pursuit gains are specific for SCA3 [26, 58, 59]. SWJ are frequently seen in SCA3, SCA6 and Friedreich Ataxia (FRDA) [26, 58] and GEN has also been observed in SCA1, SCA5, and SCA6, among many others [58]. While downbeat nystagmus is frequent in SCA27B and SCA5, it is rarely seen in SCA3 [58]. SCA6, on the other hand, can present with a hyperactive aVOR [58, 69], while aVOR-gains in SCA3 are generally decreased. A similar pattern of aVOR-gain impairment may be observed in FRDA [70], SCA1 [55], SCA6 [37], SCA7, SCA27B [71] and RFC1-related ataxia [71, 72], albeit the extent of aVOR-gain impairment and the semicircular canals affected may provide useful in the distinction. For example, isolated impairment of posterior-canal aVOR-gains favours SCA6 over SCA3 [37], and very severe aVOR-gain reductions are suggestive for RFC1-related ataxia [71, 72]. Additionally, covert catch-up saccades on vHIT appear specific for SCA3 when comparing vHIT-responses in SCA1, SCA2, SCA3, and FRDA [55].

Whereas the ocular motor and vestibular paradigms enumerated above were found to be abnormal in significant proportions of SCA3 patients, less common findings can provide useful clues for differential diagnosis. Saccadic latency, which is largely preserved in SCA3, is often increased in other ataxias [26, 58, 70]. Similarly, saccade velocity is generally normal (but can less commonly be increased or decreased). More pronounced decreases in saccade velocity are characteristic of SCA1 [26, 58], SCA7 [58, 59], and particularly SCA2 [73]. Likewise, the absence of saccadic apraxia or hyperactive head impulses further helps narrowing the differential diagnosis.

### Early Disease Detection, Measurement of Disease Progression and Grading of Disease Severity

Concerning early disease stages, pre-symptomatic carriers demonstrated fewer ocular motor abnormalities than symptomatic SCA3-patients, and only the following eye movements were reportedly abnormal: horizontal aVOR-gain [31], frequency and amplitude of SWJ [41], and AS error-rate [41]. Additionally, reflexive and volitional vertical saccades as well as the GEN slow-phase velocity differed significantly between pre-symptomatic carriers near ataxia onset (≤ 4 years from predicted ataxia onset based on CAG repeat number) and controls and sometimes deteriorated years before the predicted age of symptom-onset [27]. There were significant correlations between Z-scores of aVOR during vHIT, and reflexive vertical saccades as well as volitional vertical saccades with time to onset in pre-symptomatic carriers [27]. Furthermore, aVOR gains correlated with the time difference between the predicted age of ataxia onset based on the CAG repeat length and age for pre-symptomatic carriers, and the time difference between the age of onset and current age for symptomatic patients and was therefore described as a good biomarker of subclinical disease progression [27]. Additionally, aVOR-gain was more sensitive than the SARA score in detecting pre-symptomatic carriers [27]. Recognising these ocular motor changes at early disease stages may facilitate diagnosis and initiation of management [74].

Various ocular motor parameters demonstrated significant correlations with disease severity as specified by clinical scales such as the SARA score. This is especially true for high correlation coefficients between GEN slow-phase velocity and the SARA score in two studies with large patient cohorts [27, 42] and horizontal quantitative HIT. For aVOR-testing (by means of quantitative HIT), moderate to high inverse correlations with the SARA score were found in four [27, 31, 37, 42] out of five studies. Therefore, aVOR-gain impairment would appear to be a reliable biomarker of disease severity and progression of SCA3, as expressed in change in SARA score, especially in combination with disease duration [31]. Additionally, correlation analyses of aVOR-gain during vHIT revealed moderate to high negative correlations for NESSCA (Neurological Examination Score for Spinocerebellar Ataxia), ICARS, INAS (Inventory of Non-Ataxic Signs) count and CCFS and a moderate positive correlation with SCAFI [27] and the dizziness handicap inventory [32], enhancing its robustness and validity in assessing disease severity and disease burden.

Despite the scarcity of longitudinal data, significant correlations for monitoring disease progression could be identified in several studies [27, 28, 31]. On one hand there was a moderate negative correlation between the percentage of change of the aVOR-gain and of the SARA score [31]. On the other hand, particularly GEN slow-phase velocity and aVOR-gain revealed moderate-to-strong correlations with disease duration and estimated time to symptom-onset (in pre-symptomatic carriers) [27]. In pre-symptomatic carriers both clinical scores (NESSCA, INAS count) and aVOR-gain changes progressed significantly with decreasing time left to symptom-onset, while in symptomatic SCA3-patients this applied to all clinical scores and various ocular motor/vestibular parameters (gaze-evoked and primary-gaze nystagmus slow-phase veloicty, vertical pursuit gain and aVOR-gain) [28]. The parameters altered in pre-symptomatic carriers worsened progressively without significant age-related changes in controls [27] and more slowly than in manifest SCA3-patients [28]. Considering progression with study time, NESSCA and vertical pursuit gain worsened in pre-symptomatic subjects near (≤ 4 years from) ataxia onset, while this was not the case for pre-symptomatic carriers far (> 4 years) from predicted ataxia onset [28]. The velocity of neurological impairment as reflected by aVOR-gain and GEN slow-phase velocity in pre-symptomatic carriers was not continuous, but increased about 4 years before ataxia onset (then resembling those of symptomatic patients), whereas progression rates of volitional and reflexive vertical saccades of pre-symptomatic carriers and symptomatic patients were more similar [28]. In contrast, symptomatic SCA3 patients showed deterioration of clinical scales over time, but not of ocular motor parameters [28]. Hence the former were more sensitive to change [28], albeit less sensitive than MRI markers [75].

In conclusion, Z-scores of aVOR-gains, and reflexive and volitional vertical saccades could facilitate the prediction of disease onset. aVOR-gain, GEN slow-phase velocity and horizontal gaze-evoked eye movements have been demonstrated to be promising biomarkers of disease progression and severity as expressed by clinical scores, although both clinical scores and MRI capture changes better, while especially gaze-evoked eye movements and OKN slow-phase velocity, but also high-frequency aVOR-gains, vertical saccadic accuracy and horizontal pursuit gain were associated with disease duration. We therefore recommend measuring these parameters for disease monitoring, as described in Table 7.

**Table 7:** The value of ocular motor/vestibular parameters as biomarkers in SCA3

Clinical assessment	Parameters *
Potential markers for early onset SCA3	• Impaired vHIT gain, RVS and VVS slope [27] †
Potential markers for assessing disease duration	• Increased horizontal GEEM frequency and amplitude [41] • Decreased OKN SPV both for abduction and adduction [31] • Impaired horizontal PEM gain [41] • Decreased vertical saccadic accuracy [41] • vHIT gain reduction [42]
Potential markers for quantifying disease severity	Gaze holding: • Augmented GEN SPV [27, 42] and horizontal GEEM amplitude [41] Triggered nystagmus: • Reduced OKN SPV [42] VOR: • Impaired qHIT gain [27, 31, 32, 37, 42] Saccadic eye movements: • Increased saccade latency [45] • Decreased horizontal and vertical saccadic accuracy [41] • Augmented AS error-rate [41] • Increased RVS and VVS slope [27]
Potential markers for monitoring disease progression	• Progressive decrease in vertical PEM-gain, progressive increase in GEN SPV and SN SPV in ataxic subjects [28] • vHIT gain reduction [28, 31]

AS = anti-saccadic, DBN downbeat nystagmus, GEEM gaze-evoked eye movement, GEN gaze-evoked nystagmus, OKN optokinetic nystagmus, PEM pursuit eye movement, RVS reflexive vertical saccade slope, SCA3 spinocerebellar ataxia type 3, SN spontaneous nystagmus, SPV slow phase-velocity, SWJ square-wave jerks, VGS visually guided saccades, qHIT quantitative head-impulse testing, VOR angular vestibulo-ocular reflex, VVS volitional vertical saccade slope

* Moderate to strong correlations considered only

† Correlation between Z-scores of vHIT gain, RVS and VVS slope with time to onset in pre-symptomatic carriers

### Limitations

Several factors complicated the comparison between studies. Firstly, different measurement methods were used. Secondly, details on data retrieval were often brief or even missing. Additionally, a relatively small number of studies tested the same paradigms and limited single patient data, often impeded data synthesis. Consequently, meta-analysis was possible only for aVOR-gains [46, 53]. There is a risk that the lack of a standardised nomenclature in ocular motor measurement may have led to incorrect classification of eye movement recordings. A scarcity of longitudinal studies and information on pre-symptomatic carriers as well as a lack of treatment trials highlight the need for further research. Vergence abnormalities may be a frequent finding in SCA3 as previously reported in a critical review on ocular motor abnormalities in hereditary ataxias [58]. Importantly, the lack of quantitative data on this feature stresses the need for future studies.

## Conclusion

Frequently observed eye movement abnormalities in SCA3 patients include aVOR-gain reductions as assessed by quantitative HIT, GEN, horizontal pursuit gain decrease, dysmetric saccades, and presence of SWJ. Horizontal volitional saccade velocity and latency were without significant changes and spontaneous nystagmus was rarely observed. Information on ocular motor longitudinal assessments and early disease-stages was scarce. Nevertheless, significant correlations between GEN and OKN slow-phase velocity, high frequency aVOR-gain, horizontal pursuit gain, saccade accuracy and gaze-evoked eye movements, and disease onset, progression, and severity have been identified. Consequently, we suggest focusing primarily on quantitative assessments of eccentric gaze holding and high-frequency aVOR-gains (the latter by means of vHIT), and also consider pursuit gain, accuracy of saccades and OKN gain as potential indicators of disease severity. This systematic review has been able to provide information on parameters which may facilitate early diagnosis, monitor disease progression and provide potential treatment trial biomarkers. However, meta-analyses were not possible for most parameters due to differences or lack of clarity in measurement methods. Therefore, standardized data collection and longitudinal observational studies are required for further insights into SCA3 disease characteristics and the corresponding ocular motor and vestibular abnormalities, and to further validate their capability as potential biomarkers. With AI-based nystagmus analyses and quantitative eye tracking using consumer-based devices such as smartphones [76, 77], barriers for capturing eye movements may be substantially lowered, offering exciting opportunities to advance our understanding in rare diseases.

## Supplementary Information

Below is the link to the electronic supplementary material.

Supplementary Material 1 (DOCX 27.9 KB)

Supplementary Material 2 (DOCX 61.1 KB)

Supplementary Material 3 (DOCX 74.8 KB)

## Data Availability

The data that support the findings of this study are available from the corresponding author upon reasonable request.
