# Supplementary material for: Quantitative Ocular Motor / Vestibular Assessment in Patients with Spinocerebellar Ataxia Type 3 (SCA3, Machado Joseph Disease) – Systematic Review of the Literature
Source: Cerebellum. 2026 May 12;25(3):79. doi: 10.1007/s12311-026-02021-2 (PMC13649910; doi:10.1007/s12311-026-02021-2)
Supplement: Supplementary file 1 [file 12311_2026_2021_MOESM1_ESM.docx]

**Appendix 1: Electronic search strategy, coding-scheme for the systematic review and data analysis**

The search strategy was designed by a clinical investigator with relevant domain expertise in neurology (AAT).

We searched MEDLINE and Embase for English-language articles, using the following strategies with the following components: (1) defining the clinical syndrome (i.e., ataxia), (2) ocular motor or vestibular features, and (3) quantitative assessments. We did not expressly search for hereditary ataxia syndromes, as this was found to result in omitting most relevant studies due to lacking referral to the genetic background. We also performed a manual search of reference lists from eligible articles, and contacted corresponding authors where necessary. We did not seek to identify research abstracts from meeting proceedings or unpublished studies.

MEDLINE Search *(accessed via PubMed at [www.ncbi.nlm.nih.gov/pubmed](http://www.ncbi.nlm.nih.gov/pubmed)) on October 23^rd^ 2024 (42 citations)*

(spinocerebellar ataxia 3 OR SCA3 OR Machado Joseph disease OR ATXN3 OR ataxin 3) AND ((eye movements) OR (ocular motor) OR oculomotor OR vestibular OR saccade* OR (smooth pursuit) OR (vestibulo-ocular reflex) OR VOR OR optokinetic OR nystagmus OR gaze OR head impulse OR caloric) AND (quantitative OR recording OR recordings OR quantified OR measured OR video-oculography OR video Head Impulse Test) AND eng[la] NOT review[pt]

Embase search *on October 23^rd^ 2024 (25 citations)*

(‘spinocerebellar ataxia 3’:ab,ti OR SCA3:ab,ti OR ‘Machado Joseph disease’:ab,ti OR ATXN3:ab,ti OR ‘ataxin 3’:ab,ti) AND (‘eye movements’:ab,ti OR ‘ocular motor’:ab,ti OR oculomotor:ab,ti OR vestibular:ab,ti OR saccade* OR ‘smooth pursuit’:ab,ti OR ‘vestibulo-ocular reflex’:ab,ti OR VOR:ab,ti OR optokinetic:ab,ti OR nystagmus OR gaze OR ‘head impulse’:ab,ti OR caloric*:ab,ti) AND (quantitative:ab,ti OR recording*:ab,ti OR quantified:ab,ti OR measured:ab,ti OR ‘video Head Impulse Test’:ab,ti) AND [humans]/lim AND [English]/lim AND ([article]/lim OR [article in press]/lim)

Search Results

Our search identified 43 unique citations and 5 additional records were included after an additional literature research based on a recent systematic review on oculomotor assessment in hereditary ataxias from the same group [1]. Thus, of 48 papers screened, 6 (12.5%) were excluded at the abstract level (Figure 1, main manuscript). A record was excluded only if two scorers (IM, AAT) recommended exclusion (detailed list of predefined reasons for exclusion shown below). We did not demand concordance on reason for abstract exclusion, but, among those abstracts with concordant reasons for exclusion (12.5%, n=6), the distribution was as follows: 2 were review papers without original patient data; 1 had no data on human subjects with SCA3, 1 was not reporting on quantitative measurements and 2 were excluded for not having confirmed the disease by genetic testing.

One full text report could not be retrieved because the results had not yet been published. We therefore examined 41 full manuscripts. After initial screening, there was disagreement about study inclusion for one study between the two reviewers (IM and AAT, kappa=0.94). Overall agreement on reason for exclusion was 90.9%. We demanded concordance on reason for full-text exclusion and resolved differences by discussion.

At the end of our full-text review, 11 were excluded and 30 were considered eligible (Figure 1, main manuscript). These eligible studies represented 73.2% of the total (n=41). Among all full-text manuscripts excluded (26.8%), the distribution of reason for exclusion was as follows: 9 were not reporting on quantitative measurements and 2 had no data on human subjects with SCA3.

**Coding schema for abstract and full-text reviews**

All gathered literature was subject to title/abstract screening by two independent reviewers (IM and AAT). Full-text screening was then applied to all citations considered eligible or possibly eligible by at least one reviewer. Two independent reviewers (IM and AAT) determined whether full-text manuscripts were eligible and, if not, provided a reason for exclusion. Differences were resolved by discussion and consensus. AAT completed a hand search of the reference lists of selected articles for additional citations. For citations identified by hand search, the full process was repeated iteratively until no additional manuscripts were found for inclusion. We calculated inter-rater agreement on full-text inclusion using Cohen’s kappa [2]. A formal review protocol was not registered or posted.

Abstract Review Coding Rules

1) Coding status options are “Yes”, “No”, “Maybe”. We will review full text of “Yes” and “Maybe”. The purpose of “Yes” vs. “Maybe” is to look at kappa values agreement on “Yes” vs. “Maybe”.

2) Err on the side of “Maybe” if there is doubt about a “No”; this is more conservative.

3) If there is only a title, exclude it only if you feel confident; otherwise code it as “Maybe”.

4) Each "No" or "Maybe" should be coded with a reason for exclusion.

5) Reasons for exclusion are listed below 0-6. Go through them in order from 0 to 6 for each abstract, coding the first reason for exclusion only, not multiple reasons for exclusion. Only code "0" for “not English” if you are sure it is “not English”.

6) Two independent raters will code reason for exclusion, but we will not mandate agreement on exclusion reason at the abstract level.

7) Occasionally an abstract seems inappropriate for another reason. In such cases, code as “other”. There should be few “other” codings.

Abstract Reasons for Exclusion

| 0 | not English | manuscript is not in English |
| --- | --- | --- |
| 1 | no data | review paper; no original patient data |
| 2 | not ataxia | no data on human subjects with SCA3 / Machado Joseph disease |
| 3 | not oculomotor / vestibular | not reporting on the assessment of oculomotor and/or vestibular features |
| 4 | not quantitative | not reporting on quantitative oculomotor and/or vestibular measurements |
| 5 | abstract only | no full manuscript, but only conference abstract |
| 6 | other | any other reason abstract is not included |

Full-Text Review Coding Rules

1) Coding status options are “Yes” or “No”.

2) Each "No" should be coded with a reason for exclusion.

3) Reasons for exclusion are listed below 0-5. Go through them in order from 0 to 5 for each full text, coding the first reason for exclusion only, not multiple reasons for exclusion.

4) Two independent raters will code reason for exclusion, and we will mandate agreement on exclusion reason at the full text level.

5) Coding differences will be adjudicated or consensus will be developed through dialogue.

Full-Text Reasons for Exclusion

| 0 | not English | manuscript is not in English |
| --- | --- | --- |
| 1 | no data | review paper; no original patient data |
| 2 | abstract only | no full manuscript, but only conference abstract |
| 3 | not ataxia | no data on human subjects with SCA3 / Machado Joseph disease |
| 4 | not oculomotor / vestibular | not reporting on the assessment of oculomotor and/or vestibular features |
| 5 | not quantitative | not reporting on quantitative oculomotor and/or vestibular measurements |

**References**

[1]	Garces P, Antoniades CA, Sobanska A, Kovacs N, Ying SH, Gupta AS, et al. Quantitative Oculomotor Assessment in Hereditary Ataxia: Systematic Review and Consensus by the Ataxia Global Initiative Working Group on Digital-motor Biomarkers. Cerebellum 2024;23:896–911. https://doi.org/10.1007/s12311-023-01559-9.

[2]	Cohen J. A Coefficient of Agreement for Nominal Scales. Educational and Psychological Measurement 1960;20:37–46. https://doi.org/10.1177/001316446002000104.
