# Supplementary material for: Quantitative Ocular Motor / Vestibular Assessment in Patients with Spinocerebellar Ataxia Type 3 (SCA3, Machado Joseph Disease) – Systematic Review of the Literature
Source: Cerebellum. 2026 May 12;25(3):79. doi: 10.1007/s12311-026-02021-2 (PMC13649910; doi:10.1007/s12311-026-02021-2)
Supplement: Supplementary file 2 [file 12311_2026_2021_MOESM2_ESM.docx]

**Appendix 2 - quality assessment for studies reporting on oculomotor findings in SCA3**

Two independent reviewers (IM and AAT) rated all included studies with regards to their quality in reporting oculomotor testing in SCA3 patients based on the previously published quality assessment criteria by Garces et al. [1]. Discrepancies were resolved by discussion. Based on the eight items listed below an overall quality rating (high, moderate, low) was assigned. “High quality” was defined as having high quality on items 1, 3-7 and a low risk-of-bias for item 8. Item 2 was not considered since, depending on the study design a control group may not be required (e.g. for treatment response studies). “Moderate quality” studies were defined as studies having at least moderate quality ratings for all three items (with „low“ or „high“ risk of bias for item 8). All studies that received a low-quality rating in one or several items (items 1-7) or had an unknown risk of bias for item 8 were considered “low quality” studies.

**Criteria for the quality assessment of reporting oculomotor findings in SCA3 (from [1])**

**Study cohort related items**

1. Patient selection

high = SCA3 in all patients included was confirmed by genetic testing

moderate = no genetic testing available but either positive family history with a clear pattern of inheritance autosomal dominant [AD], autosomal recessive [AR], X-linked recessive [XR]) or established and specific diagnostic biomarkers used

low = clinically- or imaging-based diagnosis, no genetic testing, no positive family history

2. Control group selection

high = control group age-matched, independent from test group, clinical assessment (exclusion of abnormal findings) provided in detail; patients serve as their own controls in longitudinal treatment trials or treatment and placebo groups were age-matched in placebo-controlled treatment trials.

moderate = age-matched, reportedly healthy but no details reported how this was assessed

low = one or several of the following: controls are not age-matched; no information about their age is available; no information about selection process is provided; controls are genetically undetermined relatives of the test patients included.

not available = no control group is provided

**Items related to data acquisition**

3. Rigidity of recording protocol applied

high = pre-specified recording protocol available for all parameters studied, identical protocol applied in all participants and sessions

moderate = pre-specified recording protocol available only for selected parameters or different pre-specified recording protocols applied in included patients and sessions.

low = no pre-specified recording protocol used or no information provided about the use of a recording protocol.

4. Description of recording parameters used

high = all recording parameters are reported with sufficient detail to reproduce the study

moderate = recording parameters are reported partially only

low = no information provided about recording parameters

5. Appropriateness of recording devices used for ocular motor testing

high = high temporal and spatial resolution, high signal-to-noise recording technique used (e.g. search coils, high-quality video-oculography with sampling rate of >=100Hz)

moderate = recording technique with moderate to high noise levels (e.g. electro-oculography, electro-nystagmography, low to middle performance video-oculography (sampling rate <100Hz)) or behavioral paradigm used (e.g. Subjective Visual Vertical)

low = no details about recording device reported or high noise levels described

**Items related to data analysis**

6. Description of data analysis

high = data analysis is described with sufficient detail to allow reproduction of study

moderate = data analysis description is provided, but details are lacking and thus is insufficient to allow reproduction of study

low = no information provided about how the data analysis was performed or inadequate analysis pipeline

7. Assessment of obtained test results for significance

high = statistical analysis using normative values obtained from a control group or baseline values (for longitudinal studies)

moderate = statistical analysis using normative values from published literature or from the manufacturer of the device used

low = no normative values available and/or no statistical analysis performed

8. Risk of bias for assessing test results

low = index test results interpreted in a blinded fashion (i.e., without knowledge if the underlying disorder was present or absent) or automated data analysis without rater’s input.

high = index test results interpreted in a non-blinded fashion only and / or based on the rater’s subjective assessment (e.g. rating of overall pattern of vHIT traces).

unclear = no information provided about blinding of reviewers of test results.

**Table S2: quality assessment for studies reporting on oculomotor findings in SCA3– overview on all selected studies**

| Table S2: quality assessment for studies reporting on oculomotor findings in SCA3 – overview on all selected studies | | | | | | | | | |
| --- | --- | --- | --- | --- | --- | --- | --- | --- | --- |
| **Study** | **Patient selection** | **Control group selection** | **Rigidity of recording protocol applied** | **Description of recording parameters used** | **Appropriateness of recording devices used for ocular motor testing** | **Appropriateness of data analysis applied** | **Assessment of obtained test results for significance** | **Risk of bias for assessing test results** | **OVERALL study quality rating** |
| Alexandre et al. 2013 [2] | high | moderate | high | high | high | moderate | high | low | moderate |
| Bürk et al. 1996 [3] | high | moderate | high | moderate | moderate | high | high | unclear | low |
| Buttner et al. 1998 [4] | high | not applicable | high | high | moderate | high | moderate | high | moderate |
| Caspi et al. 2013 [5] | high | moderate | high | high | high | high | high | high | low |
| de Oliveira et al. 2021 [6] | high | low | high | moderate | high | moderate | high | low | low |
| de Oliveira et al. 2023 [7] | high | low | high | moderate | high | moderate | high | low | low |
| Durig et al. 2002 [8] | high | not applicable | high | moderate | moderate | low | low | unclear | low |
| Elyoseph et al. 2023 [9] | high | moderate | high | high | high | high | moderate | low | moderate |
| Elyoseph et al. 2023 [10] | high | moderate | high | high | high | high | moderate | low | moderate |
| Elyoseph et al. 2024 [11] | high | high | high | moderate | high | high | moderate | low | moderate |
| Elyoseph et al. 2024 [12] | high | moderate | high | high | high | high | moderate | low | moderate |
| Geisinger et al. 2021 [13] | high | high | high | high | high | high | moderate | low | high |
| Ghasia et al. 2016 [14] | high | not applicable | high | moderate | high | high | low | high | low |
| Gordon et al. 2003 [15] | high | not applicable | high | high | low | low | low | unclear | low |
| Gordon et al. 2014 [16] | high | moderate | high | high | high | high | high | low | high |
| Kim et al. 2023 [17] | high | moderate | high | moderate | moderate | moderate | high | low | moderate |
| Kim et al. 2013 [18] | high | moderate | high | high | high | moderate | high | high | moderate |
| Kim et al. 2021 [19] | high | not applicable | high | low | low | low | low | unclear | low |
| Lemos et al. 2021 [20] | high | not applicable | high | high | high | low | low | unclear | low |
| Lemos et al. 2018 [21] | high | moderate | high | high | high | high | high | low | high |
| Luis et al. 2016 [22] | high | high | high | high | high | high | high | low | high |
| Ohyagi et al. 2000 [23] | high | low | high | moderate | * | low | low | low | low |
| Ribeiro et al. 2015 [24] | high | moderate | high | high | * | high | high | low | high |
| Takegoshi et al. 2000 [25] | high | low | low | high | moderate | high | high | low | low |
| Wu et al. 2017 [26] | high | high | high | high | high | high | high | low | high |
| Bürk et al. 1997 [27] | high | moderate | high | moderate | moderate | high | high | low | low |
| Chang et al. 2020 [28] | high | low | high | high | moderate | high | high | low | low |
| Crane et al. 2000 [29] | high | low | high | high | high | high | high | low | low |
| Wessel et al. 1998 [30] | high | moderate | high | high | moderate | high | high | low | low |
| Gordon et al. 2008 [31] | high | low | high | high | high | moderate | high | unclear | low |

* In these studies [23,24], only myographies, but no eye movement recordings were performed. Thus, the appropriateness of the recording devices was not determined.

**References**

[1]	Garces P, Antoniades CA, Sobanska A, Kovacs N, Ying SH, Gupta AS, et al. Quantitative Oculomotor Assessment in Hereditary Ataxia: Discriminatory Power, Correlation with Severity Measures, and Recommended Parameters for Specific Genotypes. Cerebellum 2024;23:121–35. https://doi.org/10.1007/s12311-023-01514-8.

[2]	Alexandre MF, Rivaud-Péchoux S, Challe G, Durr A, Gaymard B. Functional Consequences of Oculomotor Disorders in Hereditary Cerebellar Ataxias. Cerebellum 2013;12:396–405. https://doi.org/10.1007/s12311-012-0433-z.

[3]	Bürk K, Abele M, Fetter M, Dichgans J, Skalej M, Laccone F, et al. Autosomal dominant cerebellar ataxia type I Clinical features and MRI in families with SCA1, SCA2 and SCA3. Brain 1996;119:1497–505. https://doi.org/10.1093/brain/119.5.1497.

[4]	Buttner N, Geschwind D, Jen JC, Perlman S, Pulst SM, Baloh RW. Oculomotor phenotypes in autosomal dominant ataxias. Arch Neurol 1998;55:1353–7. https://doi.org/10.1001/archneur.55.10.1353.

[5]	Caspi A, Zivotofsky AZ, Gordon CR. Multiple Saccadic Abnormalities in Spinocerebellar Ataxia Type 3 Can Be Linked to a Single Deficiency in Velocity Feedback. Invest Ophthalmol Vis Sci 2013;54:731. https://doi.org/10.1167/iovs.12-10689.

[6]	De Oliveira CM, Leotti VB, Bolzan G, Cappelli AH, Rocha AG, Ecco G, et al. Pre‐ataxic Changes of Clinical Scales and Eye Movement in Machado–Joseph Disease: BIGPRO Study. Movement Disorders 2021;36:985–94. https://doi.org/10.1002/mds.28466.

[7]	De Oliveira CM, Leotti VB, Cappelli AH, Rocha AG, Ecco G, Bolzan G, et al. Progression of Clinical and Eye Movement Markers in Preataxic Carriers of Machado‐Joseph Disease. Movement Disorders 2023;38:26–34. https://doi.org/10.1002/mds.29226.

[8]	Durig JS, Jen JC, Demer JL. Ocular motility in genetically defined autosomal dominant cerebellar ataxia. Am J Ophthalmol 2002;133:718–21. https://doi.org/10.1016/s0002-9394(02)01328-4.

[9]	Elyoseph Z, Geisinger D, Zaltzman R, Gordon CR, Mintz M. How vestibular dysfunction transforms into symptoms of depersonalization and derealization? Journal of the Neurological Sciences 2023;444. https://doi.org/10.1016/j.jns.2022.120530.

[10]	Elyoseph Z, Geisinger D, Zaltzman R, Hartman TG, Gordon CR, Mintz M. The overarching effects of vestibular deficit: Imbalance, anxiety, and spatial disorientation. Journal of the Neurological Sciences 2023;451. https://doi.org/10.1016/j.jns.2023.120723.

[11]	Elyoseph Z, Geisinger D, Zaltzman R, Mintz M, Gordon CR. Horizontal Vestibulo-Ocular Reflex Deficit as a Biomarker for Clinical Disease Onset, Severity, and Progression of Machado-Joseph Disease. The Cerebellum 2024;23:1273–9. https://doi.org/10.1007/s12311-023-01552-2.

[12]	Elyoseph Z, Geisinger D, Zaltzman R, Mintz M, Gordon CR. The vestibular symptomatology of Machado-Joseph Disease. VES 2024;34:159–67. https://doi.org/10.3233/VES-230038.

[13]	Geisinger D, Elyoseph Z, Zaltzman R, Mintz M, Gordon CR. Angular vestibulo ocular reflex loss with preserved saccular function in Machado-Joseph disease. Journal of the Neurological Sciences 2021;424. https://doi.org/10.1016/j.jns.2021.117393.

[14]	Ghasia FF, Wilmot G, Ahmed A, Shaikh AG. Strabismus and Micro-Opsoclonus in Machado-Joseph Disease. Cerebellum 2016;15:491–7. https://doi.org/10.1007/s12311-015-0718-0.

[15]	Gordon CR, Joffe V, Vainstein G, Gadoth N. Vestibulo-ocular arreflexia in families with spinocerebellar ataxia type 3 (Machado-Joseph disease). Journal of Neurology, Neurosurgery & Psychiatry 2003;74:1403–6. https://doi.org/10.1136/jnnp.74.10.1403.

[16]	Gordon CR, Zivotofsky AZ, Caspi A. Impaired vestibulo-ocular reflex (VOR) in spinocerebellar ataxia type 3 (SCA3): Bedside and search coil evaluation. VES 2014;24:351–5. https://doi.org/10.3233/VES-140527.

[17]	Kim JM, Nam TS, Choi SM, Kim BC, Lee SH. Clinical value of vestibulo-ocular reflex in the differentiation of spinocerebellar ataxias. Sci Rep 2023;13:14783. https://doi.org/10.1038/s41598-023-41924-6.

[18]	Kim JS, Kim JS, Youn J, Seo D, Jeong Y, Kang J, et al. Ocular motor characteristics of different subtypes of spinocerebellar ataxia: Distinguishing features. Movement Disorders 2013;28:1271–7. https://doi.org/10.1002/mds.25464.

[19]	Kim M, Ahn JH, Mun JK, Choi EH, Kim JS, Youn J, et al. Extracerebellar Signs and Symptoms in 117 Korean Patients with Early-Stage Spinocerebellar Ataxia. J Clin Neurol 2021;17:242. https://doi.org/10.3988/jcn.2021.17.2.242.

[20]	Lemos J, Novo A, Duque C, Cunha I, Ribeiro J, Castelhano J, et al. Static and Dynamic Ocular Motor Abnormalities as Potential Biomarkers in Spinocerebellar Ataxia Type 3. Cerebellum 2021;20:402–9. https://doi.org/10.1007/s12311-020-01217-4.

[21]	Lemos J, Novo A, Duque C, Castelhano J, Eggenberger E, Januário C. “Pinball” intrusions in spinocerebellar ataxia type 3. Neurology 2018;90:36–7. https://doi.org/10.1212/WNL.0000000000004772.

[22]	Luis L, Costa J, Muñoz E, De Carvalho M, Carmona S, Schneider E, et al. Vestibulo-ocular reflex dynamics with head-impulses discriminates spinocerebellar ataxias types 1, 2 and 3 and Friedreich ataxia. VES 2016;26:327–34. https://doi.org/10.3233/VES-160579.

[23]	Ohyagi Y, Yamada T, Okayama A, Sakae N, Yamasaki T, Ohshima T, et al. Vergence disorders in patients with spinocerebellar ataxia 3/Machado- Joseph disease: A synoptophore study. J Neurol Sci 2000;173:120–3. https://doi.org/10.1016/S0022-510X(99)00309-3.

[24]	Ribeiro RS, Pereira MM, Pedroso JL, Braga-Neto P, Barsottini OGP, Manzano GM. Cervical and ocular vestibular evoked potentials in Machado-Joseph disease: Functional involvement of otolith pathways. J Neurol Sci 2015;358:294–8. https://doi.org/10.1016/j.jns.2015.09.013.

[25]	Takegoshi H, Murofushi T. Vestibular Evoked Myogenic Potentials in Patients with Spinocerebellar Degeneration. Acta Oto-Laryngologica 2000;120:821–4. https://doi.org/10.1080/000164800750061660.

[26]	Wu C, Chen DB, Feng L, Zhou XX, Zhang JW, You HJ, et al. Oculomotor deficits in spinocerebellar ataxia type 3: Potential biomarkers of preclinical detection and disease progression. CNS Neurosci Ther 2017;23:321–8. https://doi.org/10.1111/cns.12676.

[27]	Bürk K, Fetter M, Skalej M, Laccone F, Stevanin G, Dichgans J, et al. Saccade velocity in idiopathic and autosomal dominant cerebellar ataxia. J Neurol Neurosurg Psychiatry 1997;62:662–4.

[28]	Chang Z, Chen Z, Stephen CD, Schmahmann JD, Wu HT, Sapiro G, et al. Accurate detection of cerebellar smooth pursuit eye movement abnormalities via mobile phone video and machine learning. Sci Rep 2020;10:18641. https://doi.org/10.1038/s41598-020-75661-x.

[29]	Crane BT, Tian JR, Demer JL. Initial vestibulo-ocular reflex during transient angular and linear acceleration in human cerebellar dysfunction. Experimental Brain Research 2000;130:486–96. https://doi.org/10.1007/s002219900266.

[30]	Wessel K, Moschner C, Wandinger KP, Kömpf D, Heide W. Oculomotor Testing in the Differential Diagnosis of Degenerative Ataxic Disorders. Arch Neurol 1998;55:949. https://doi.org/10.1001/archneur.55.7.949.

[31]	Gordon CR, Caspi A, Levite R, Zivotofsky AZ. Mechanisms of vestibulo-ocular reflex (VOR) cancellation in spinocerebellar ataxia type 3 (SCA-3) and episodic ataxia type 2 (EA-2). Progress in Brain Research, vol. 171, Elsevier; 2008, p. 519–25. https://doi.org/10.1016/S0079-6123(08)00674-2.
