# Supplementary material for: Quantitative Ocular Motor / Vestibular Assessment in Patients with Spinocerebellar Ataxia Type 3 (SCA3, Machado Joseph Disease) – Systematic Review of the Literature
Source: Cerebellum. 2026 May 12;25(3):79. doi: 10.1007/s12311-026-02021-2 (PMC13649910; doi:10.1007/s12311-026-02021-2)
Supplement: Supplementary file 3 [file 12311_2026_2021_MOESM3_ESM.docx]

**Appendix 3: Correlations: Oculomotor / vestibular parameters and scores (Spearman)**

|  | **SARA** | **DHI** | **ABC** | **VVS** | **NESSCA** | **ICARS** | **INAS count** | **SCAFI** | **CCFS** |
| --- | --- | --- | --- | --- | --- | --- | --- | --- | --- |
| **Nystagmus** |  |  |  |  |  |  |  |  |  |
| Central nystagmus SPV | -0.144 [1] ac |  |  |  | **0.347**** [1] b | **0.299*** [1] b | 0.200 [1] b | **-0.323**** [1] b | **0.311*** [1] b |
| GEN SPV | **0.686**** [1] ac, abduction **0.716**** [2] ac, adduction: **0.626**** [2] ac |  |  |  | **0.676**** [1] b | **0.764**** [1] b | **0.495**** [1] b | **-0.643**** [1] b | **0.629**** [1] b |
| Hor GEEM frequency (including SWJ and GEN) | **0.593**** [3] § b |  |  |  |  |  |  |  |  |
| Hor GEEM amplitude | **0.760**** [3] § b |  |  |  |  |  |  |  |  |
| OKN SPV | abduction **-0.659**** [2] ac, adduction **-0.521**** [2] ac |  |  |  |  |  |  |  |  |
| **Saccadic intrusions** |  |  |  |  |  |  |  |  |  |
| SWJ frequency | **0.319*** [3] § b |  |  |  |  |  |  |  |  |
| SWJ amplitude | **0.287*** [3] § b |  |  |  |  |  |  |  |  |
| **VOR** |  |  |  |  |  |  |  |  |  |
| Hor high frequency VOR (vHIT) | **-0.454*** [1] ac, **-0.81**** [4] b † ‡, **-0.53**** [4] ac, -0.48 [5] ac, **-0.900*** [6] ac, abduction: **-0.592**** [2] ac, adduction: **-0.718**** [2] ac | **0.49*** [7] ac | NS [7] ac | NS [7] ac | **-0.709**** [1] b | **-0.733**** [1] b | **-0.533**** [1] b | **0.673**** [1] § b | **-0.572**** [1] § b |
| AC gain | -0.200 [6] ac |  |  |  |  |  |  |  |  |
| PC gain | -0.359 [6] ac |  |  |  |  |  |  |  |  |
| AC/PC ratio | 0.300 [6] ac |  |  |  |  |  |  |  |  |
| **Pursuit eye movements** |  |  |  |  |  |  |  |  |  |
| Hor pursuit gain | abduction: **-0.477**** [2] ac, adduction: **-0.328*** [2] ac, **-0.642**** [3] § b |  |  |  |  |  |  |  |  |
| Vert pursuit gain | -0.289 [1] ac, **-0.347**** [3] § b |  |  |  | -0.314 [1] b | **-0.381**** [1] b | -0.184 [1] b | **0.363**** [1] § b | **-0.390**** [1] § b |
| **Saccadic eye movements** |  |  |  |  |  |  |  |  |  |
| RV saccade slope (PD vs amplitude) | **0.416*** [1] ac |  |  |  | **0.447**** [1] b | **0.515**** [1] b | **0.288*** [1] b | **-0.479**** [1] b | **0.388**** [1] b |
| VV saccade slope (PD vs amplitude) | 0.008 [1] ac |  |  |  | **0.482**** [1] b | **0.439**** [1] b | **0.389**** [1] b | **-0.403**** [1] b | **0.406**** [1] b |
| Hor saccades PV | **-0.345**** [3] § b |  |  |  |  |  |  |  |  |
| Vert saccades PV | **-0.397**** [3] § b |  |  |  |  |  |  |  |  |
| Saccade latency | **0.73**** [8] ac**, 0.360**** [3] § b |  |  |  |  |  |  |  |  |
| Hor saccadic accuracy | **-0.480**** [3] § b |  |  |  |  |  |  |  |  |
| Vert saccadic accuracy | **-0.547**** [3] § b |  |  |  |  |  |  |  |  |
| Antisaccadic error rate | **0.533**** [3] § b |  |  |  |  |  |  |  |  |

*Abbreviations* ABC = Activities specific Balance Confidence Scale; AC = anterior canal; CCFS = Composite Cerebellar Function Score; DHI = Dizziness Handicap Inventory; GEEM = gaze-evoked eye movements; GEN = gaze-evoked nystagmus; HC = horizontal canal; hor = horizontal; ICARS = International Cooperative Ataxia Rating Scale; INAS = Inventory of Non-Ataxic Signs; NESSCA = Neurological examination Score for Spinocerebellar Ataxia; OKN = optokinetic nystagmus; PC = posterior canal; PD = peak duration; PV = peak velocity; RVS = reflexive vertical saccade; SARA = Scale for Assessment and Rating of Ataxia; SCAFI = Spinocerebellar Ataxia Functional Index; SWJ = square wave jerks; SPV = slow-phase velocity; vHIT = video head impulse testing; VEMP, vert = vertical; VOR = vestibulo-ocular reflex; VVS = volitional vertical saccade

*Labeling of disease stage:*

ac = ataxic carriers, pac = pre-ataxic carriers, b=both ataxic and pre-ataxic carriers

*/** values in bold are significant (* p≤0.05, **p≤0.01)

† Both pre-ataxic and ataxic carriers and controls.

‡ Additional Pearson correlation analyses between horizontal VOR gain and SARA score in 20 patients in two examinations (first examination: r=-0.66**, p<0.001; second examination=-0.61**, p<0.001) and between change in percent of the VOR gain and change in percent of the SARA score in 20 patients across two examinations (r=**-0.54*** ac, p<0.05)

§ Pearson correlation analysis

**Correlation: Oculomotor / vestibular parameters and patient characteristics (Spearman)**

|  | **CAG repeat length** | **Age at onset** | **Disease duration** | **Age** | **Correlation between Z-scores and time to onset in pre-ataxic carriers** | **Time to / time after onset of pre-ataxic and ataxic carriers** | **Time to / time after ataxia onset (using predicted age of ataxia onset at birth)** |
| --- | --- | --- | --- | --- | --- | --- | --- |
| **Nystagmus** |  |  |  |  |  |  |  |
| Central nystagmus SPV |  |  |  | 0.083 [1] pac |  | **0.331**** [1] pac | **0.276*** [1] b |
| GEN SPV | abduction: **0.395*** [2] ac; adduction: **0.432**** [2] ac |  |  | 0.083 [1] pac |  | **0.724**** [1] pac | **0.667**** [1] b |
| Hor GEEM frequency (including SWJ and GEN) | NS [3] † b |  | **0.550**** [3] † b |  |  |  |  |
| Hor GEEM amplitude | NS [3] † b |  | **0.526**** [3] † b |  |  |  |  |
| OKN SPV | abduction: **-0.504**** [2] ac, adduction: **-0.543**** [2] |  | abduction: **-0.542**** [2] ac, adduction **-0.450**** [2] ac |  |  |  |  |
| **Saccadic intrusions** |  |  |  |  |  |  |  |
| SWJ frequency | NS [3] † b |  | NS [3] † b |  |  |  |  |
| SWJ amplitude | NS [3] † b |  | NS [3] † b |  |  |  |  |
| **VOR** |  |  |  |  |  |  |  |
| Hor high frequency VOR (vHIT) | -**0.35*** [4] † ac, -0.429 [6] ac, abduction: **-0.520**** [2] ac, adduction: **-0.588**** [2] ac | 0.771 [6] ac | -0.304 [6] ac, abduction: **-0.460**** [2] ac; adduction: **-0.427**** [2] ac | -0.388 [1] † pac | **-0.516**** [1] † pac | **-0.735**** [1] pac | **-0.725**** [1] b |
| AC gain | -0.086 [6] ac | 0.086 [6] ac | -0.493 [6] ac |  |  |  |  |
| PC gain | -0.580 [6] ac | 0.493 [6] ac | -0.485 [6] ac |  |  |  |  |
| AC/PC ratio | 0.771 [6] ac | -0.771 [6] ac | 0.174 [6] ac |  |  |  |  |
| **Number of abnormal VEMP responses** ‡ |  |  | 0.52 [9] ac | 0.00 [9] ac |  |  |  |
| **Pursuit eye movments** |  |  |  |  |  |  |  |
| Hor pursuit gain | NS [3] † b |  | **-0.470**** [3] b |  |  |  |  |
| Vert pursuit gain | NS [3] † b |  | NS [3] † b | -0.101 [1] † pac | -0.226 [1] † pac | **-0.364**** [1] pac | **-0.262*** [1] b |
| **Saccadic eye movements** |  |  |  |  |  |  |  |
| RV saccade slope (PD vs amplitude) |  |  |  | -0.010 [1] pac | **0.411*** [1] pac | **0.490**** [1] pac | **0.505**** [1] b |
| VV saccade slope (PD vs amplitude) |  |  |  | 0.229 [1] pac | **0.561**** [1] pac | **0.510**** [1] pac | **0.523**** [1] b |
| Hor saccades PV | NS [3] † b |  | NS [3] † b |  |  |  |  |
| Vert saccades PV | NS [3] † b |  | **-0.282*** [3] † b |  |  |  |  |
| Saccade latency | NS [3] † b |  | **0.280*** [3] † b |  |  |  |  |
| Hor saccadic accuracy | NS [3] † b |  | **-0.327*** [3] † b |  |  |  |  |
| Vert saccadic accuracy | NS [3] † b |  | **-0.471**** [3] † b |  |  |  |  |
| Antisaccadic error rate | **0.330**** [3] † b |  | **0.360**** [3] † b |  |  |  |  |

*Abbreviations* AC = anterior canal; GEEM = gaze-evoked eye movements; GEN = gaze-evoked nystagmus; hor = horizontal; OKN = optokinetic nystagmus; PC = posterior canal; PD = peak duration; PV = peak velocity; RV = reflexive vertical; SWJ = square wave jerks; SPV = slow-phase velocity; vHIT = video head impulse testing; VEMP = vestibular evoked myogenic potential; vert = vertical; VOR = vestibulo-ocular reflex; VV = volitional vertical; VVS = Vertigo Visual Scale

*Labeling of disease stage:*

ac = ataxic carriers, pac = pre-ataxic carriers, b=both ataxic and pre-ataxic carriers

*/** values in bold are significant (* p≤0.05, **p≤0.01)

† Pearson correlation analysis

‡ Including cervical and ocular vestibular evoked myogenic potential responses

[1]	De Oliveira CM, Leotti VB, Bolzan G, Cappelli AH, Rocha AG, Ecco G, et al. Pre‐ataxic Changes of Clinical Scales and Eye Movement in Machado–Joseph Disease: BIGPRO Study. Movement Disorders 2021;36:985–94. https://doi.org/10.1002/mds.28466.

[2]	Lemos J, Novo A, Duque C, Cunha I, Ribeiro J, Castelhano J, et al. Static and Dynamic Ocular Motor Abnormalities as Potential Biomarkers in Spinocerebellar Ataxia Type 3. Cerebellum 2021;20:402–9. https://doi.org/10.1007/s12311-020-01217-4.

[3]	Wu C, Chen DB, Feng L, Zhou XX, Zhang JW, You HJ, et al. Oculomotor deficits in spinocerebellar ataxia type 3: Potential biomarkers of preclinical detection and disease progression. CNS Neurosci Ther 2017;23:321–8. https://doi.org/10.1111/cns.12676.

[4]	Elyoseph Z, Geisinger D, Zaltzman R, Mintz M, Gordon CR. Horizontal Vestibulo-Ocular Reflex Deficit as a Biomarker for Clinical Disease Onset, Severity, and Progression of Machado-Joseph Disease. The Cerebellum 2024;23:1273–9. https://doi.org/10.1007/s12311-023-01552-2.

[5]	Gordon CR, Zivotofsky AZ, Caspi A. Impaired vestibulo-ocular reflex (VOR) in spinocerebellar ataxia type 3 (SCA3): Bedside and search coil evaluation. VES 2014;24:351–5. https://doi.org/10.3233/VES-140527.

[6]	Kim JM, Nam TS, Choi SM, Kim BC, Lee SH. Clinical value of vestibulo-ocular reflex in the differentiation of spinocerebellar ataxias. Sci Rep 2023;13:14783. https://doi.org/10.1038/s41598-023-41924-6.

[7]	Elyoseph Z, Geisinger D, Zaltzman R, Mintz M, Gordon CR. The vestibular symptomatology of Machado-Joseph Disease. VES 2024;34:159–67. https://doi.org/10.3233/VES-230038.

[8]	Alexandre MF, Rivaud-Péchoux S, Challe G, Durr A, Gaymard B. Functional Consequences of Oculomotor Disorders in Hereditary Cerebellar Ataxias. Cerebellum 2013;12:396–405. https://doi.org/10.1007/s12311-012-0433-z.

[9]	Ribeiro RS, Pereira MM, Pedroso JL, Braga-Neto P, Barsottini OGP, Manzano GM. Cervical and ocular vestibular evoked potentials in Machado-Joseph disease: Functional involvement of otolith pathways. J Neurol Sci 2015;358:294–8. https://doi.org/10.1016/j.jns.2015.09.013.
